# Supplementary material for: Enhancing plasticity in central networks improves motor and sensory recovery after nerve damage
Source: Nat Commun. 2019 Dec 19;10:5782. doi: 10.1038/s41467-019-13695-0 (PMC6923364; doi:10.1038/s41467-019-13695-0)
Supplement: Supplementary file 1 — Supplementary Information [file 41467_2019_13695_MOESM1_ESM.pdf]

Enhancing plasticity in central networks improves motor and sensory recovery after nerve damage

Meyers et al.

Supplementary Information:

Supplementary Figure 1. Performance on the isometric pull task.

Supplementary Figure 2. VNS does not alter the total size or excitability of motor cortex

Supplementary Figure 3. Bubble plots detailing the cortical locations of digit flexion, wrist extension, and digit flexion + elbow movements.

Supplementary Figure 4. VNS does not change stimulation current thresholds required to evoke multi-joint movements.

Supplementary Figure 5. Analysis of success rate and pull force on the isometric pull task with individual rats depicted.

Supplementary Figure 6. CL-VNS improves recovery of speed of force generation.

Supplementary Figure 7. Distribution of pull forces.

Supplementary Figure 8. Differences in the intensity of rehabilitative training or number of stimulations cannot account for recovery.

Supplementary Figure 9. Reversal of maladaptive plasticity restores sensory function after nerve injury.

Supplementary Figure 10. Morphological analysis of injured median nerve.

Supplementary Figure 11. Morphological analysis of injured ulnar nerve.

Supplementary Figure 12. Scatter plots of G-ratio as a function of fiber area in the median nerve both proximal and distal to injury site.

Supplementary Figure 13. Scatter plots of the G-ratio as a function of fiber area in the ulnar nerve both proximal and distal to injury site.

Supplementary Figure 14. Muscle fiber density in the flexor digitorum profundus (FDP) that was denervated by the nerve injury.

Supplementary Figure 15. VNS enhances synaptic reorganization in central networks after peripheral nerve damage.

Supplementary Figure 16. Size and excitability of motor cortex in Experiment 2.

Supplementary Figure 17. Cortical cholinergic depletion does not prevent motor recovery during rehabilitation.

Supplementary Figure 18. Distribution of pull forces in Experiment 2.

Supplementary Figure 19. Additional metrics of isometric pull task performance in the second set of experiments.

Supplementary Figure 20. Reversal of maladaptive plasticity is necessary to restore sensory function after nerve injury.

Supplementary Table 1. Experiment 1: Success rates on isometric pull task for all animals during isometric pull training

Supplementary Table 2. Experiment 1: Pull forces on isometric pull task for all animals during isometric pull training.

Supplementary Table 3. Experiment 1: Von Frey sensory thresholds.

Supplementary Table 4. Experiment 1: Cylinder forelimb asymmetry index (%).

Supplementary Table 5. Morphological analysis values of median nerve.

Supplementary Table 6. Morphological analysis values of ulnar nerve.

Supplementary Table 7. Muscle fiber area and density.

Supplementary Table 8. Experiment 1: Cortical area for intracortical microstimulation evoked movements.

Supplementary Table 9. Cortical PRV-positive cell counts.

Supplementary Table 10. Experiment 2: Success rate on the isometric pull task for all animals during isometric pull training.

Supplementary Table 11. Experiment 2: Pull forces on the isometric pull task for all animals during isometric pull training.

Supplementary Table 12. Experiment 2: Percent of cortical cholinergic innervation depleted following lesions of the nucleus basalis.

Supplementary Table 13. Experiment 2: Cortical area for intracortical microstimulation evoked movements.

Supplementary Table 14. Experiment 2: Von Frey sensory thresholds

Supplementary Table 15. Experiment 2: Cylinder forelimb asymmetry index (%).

Supplementary Table 16. Statistical values for the morphological analysis of median nerve.

Supplementary Table 17. Statistical values for the morphological analysis of ulnar nerve.

Supplementary Table 18. ANOVAs and Kruskal-Wallis tests for all figures.

Supplementary Table 19. Means and variance for all figures.

Supplementary Table 20. Statistical tests for all figures.

Supplementary Table 21. Cuff impedance for subjects at Week 12.

Supplementary Information:

I. Supplementary Figures

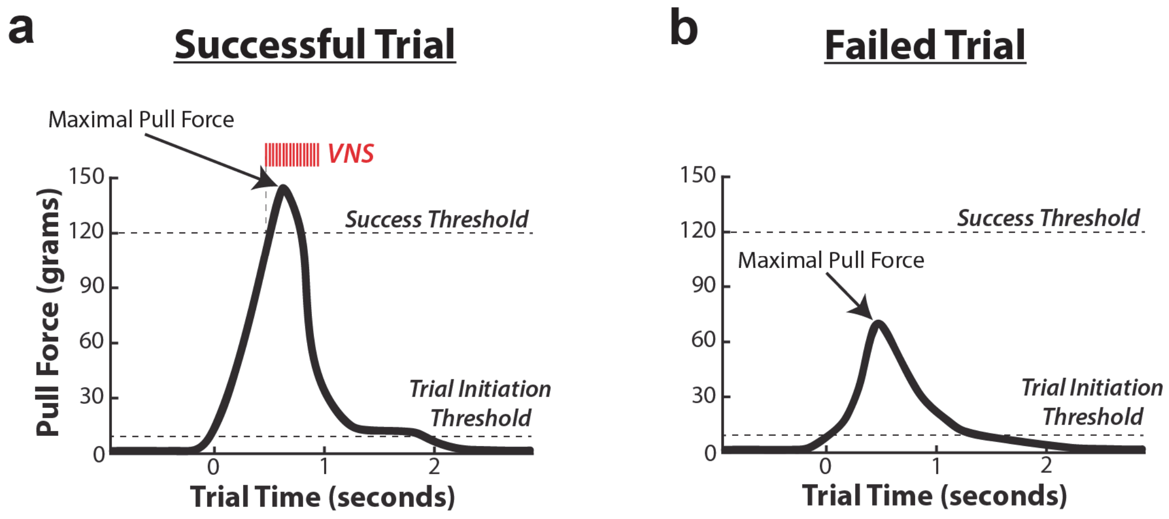

Supplementary Figure 1. Performance on the isometric pull task. (a) A single representative trial illustrating a successful pull attempt. The upper horizontal black dashed line indicates the pull force success threshold, and the lower dashed line indicates the trial initiation threshold. Immediately upon success threshold crossing VNS was delivered if applicable. (b) A representative trial illustrating an unsuccessful pull attempt in which the force signal did not cross the success threshold, thus no pellet reward or VNS was delivered.

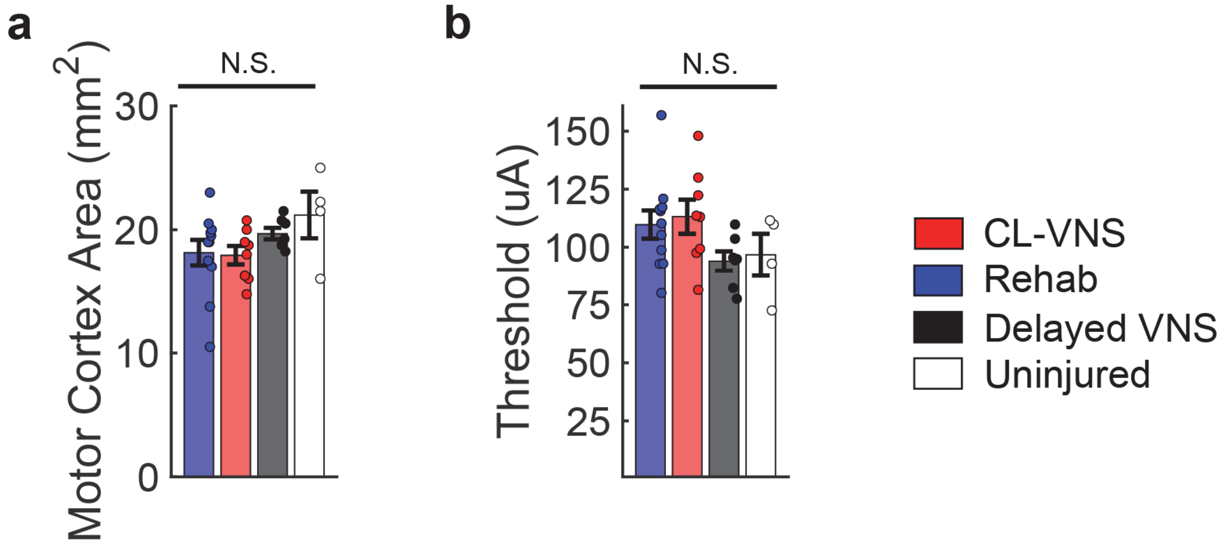

Supplementary Figure 2. VNS does not alter the total size or excitability of motor cortex. No difference was observed in (a) ICMS evoked motor cortex area (One-way ANOVA, $F[3,29]=1.68$, $p=0.20$) or (b) ICMS thresholds to evoke movements (One-way ANOVA, $F[3,29]=1.86$, $p=0.16$). Circles depict individual subjects. Error bars indicate S.E.M. *** $p < 0.001$, ** $p < 0.01$, * $p < 0.05$, N.S.: not significant. Source data are provided as a Source Data file.

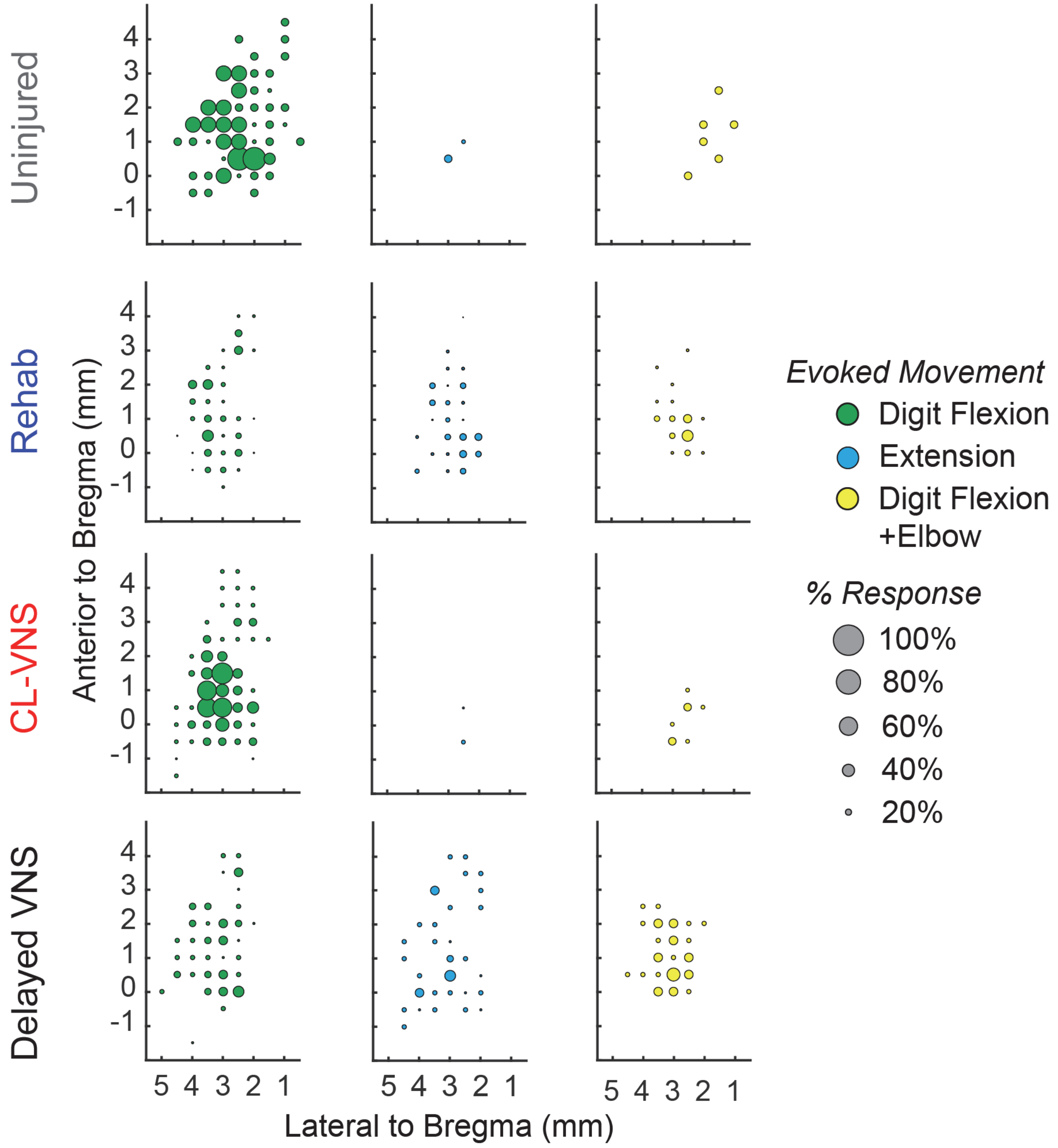

Supplementary Figure 3. Bubble plots detailing the cortical locations of digit flexion, wrist extension, and digit flexion + elbow movements. Extension of Figure 2 with the Uninjured data shown here.

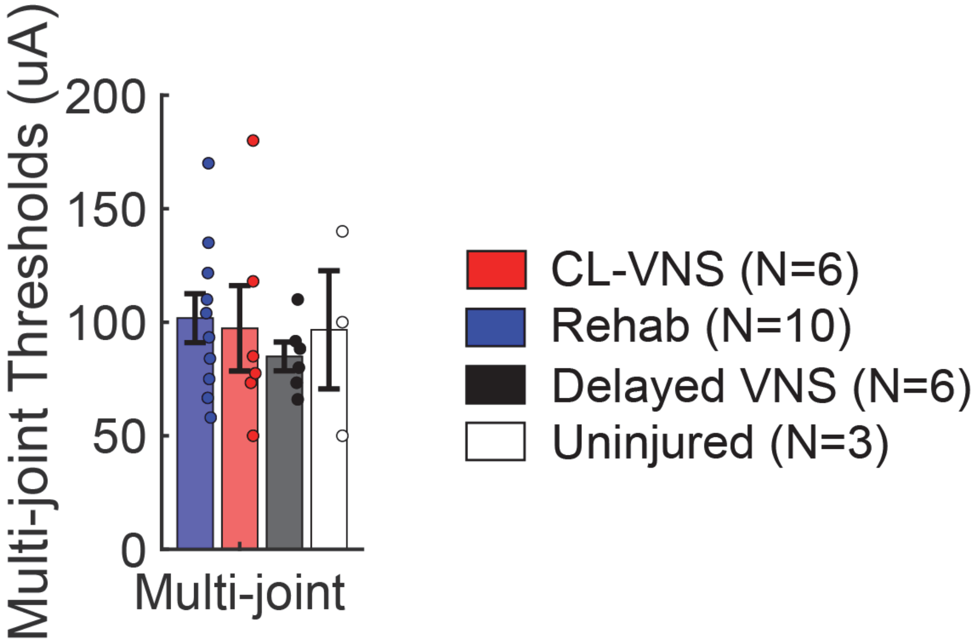

Supplementary Figure 4. VNS does not change stimulation current thresholds required to evoke multi-joint movements. No differences were observed across groups for the ICMS stimulation current intensity required to evoke multi-joint movements (One-way ANOVA, $F[3,24]=0.29$, $p=0.83$; Rehab: n=10, CL-VNS: n=6, Delayed VNS: n=6, Uninjured: n=3). Refer to Figure 2c. Circles depict individual subjects. Error bars indicate S.E.M. *** $p < 0.001$, ** $p < 0.01$, * $p < 0.05$, N.S.: not significant. Source data are provided as a Source Data file.

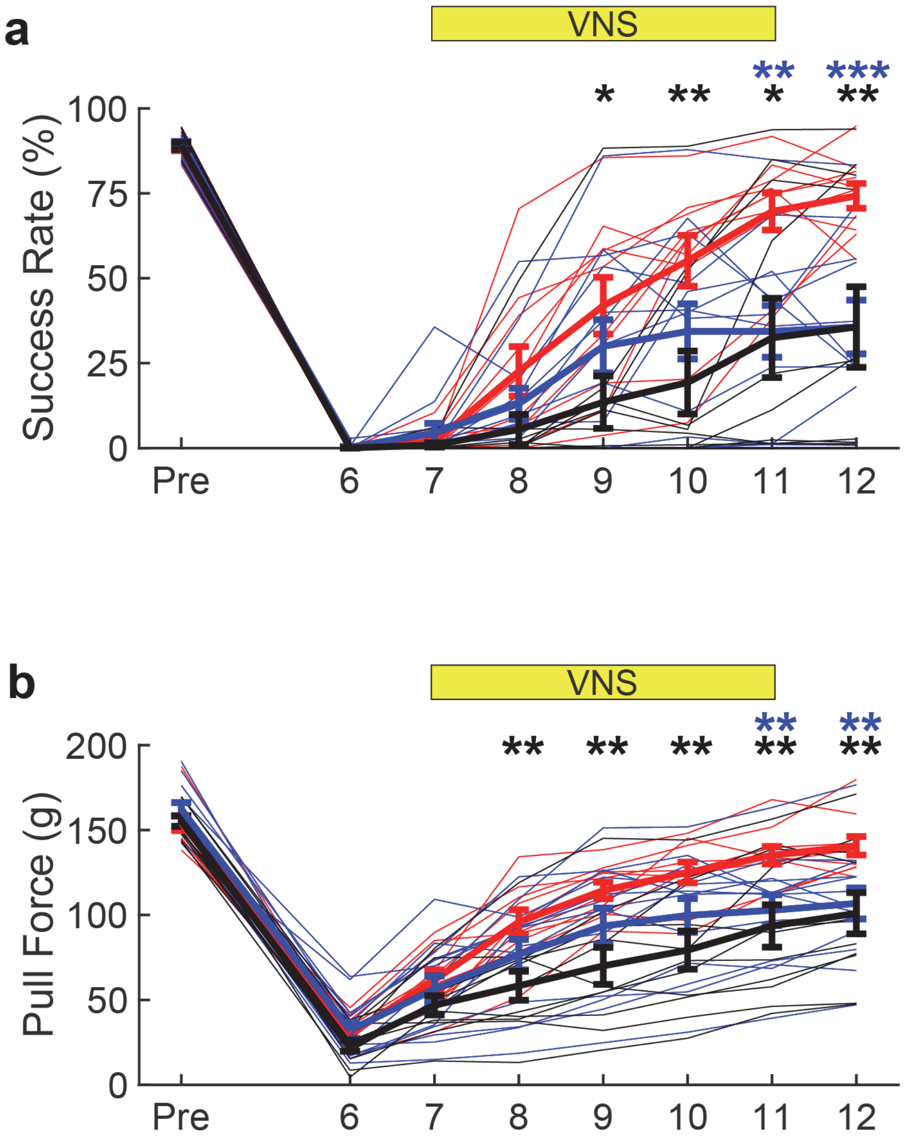

Supplementary Figure 5. Analysis of success rate and pull force on the isometric pull task with individual rats depicted. Compare to Figure 3. (a) Success rate and (b) pull force with individual animals shown. Error bars indicate S.E.M. Thin lines depict individual subjects. Source data are provided as a Source Data file.

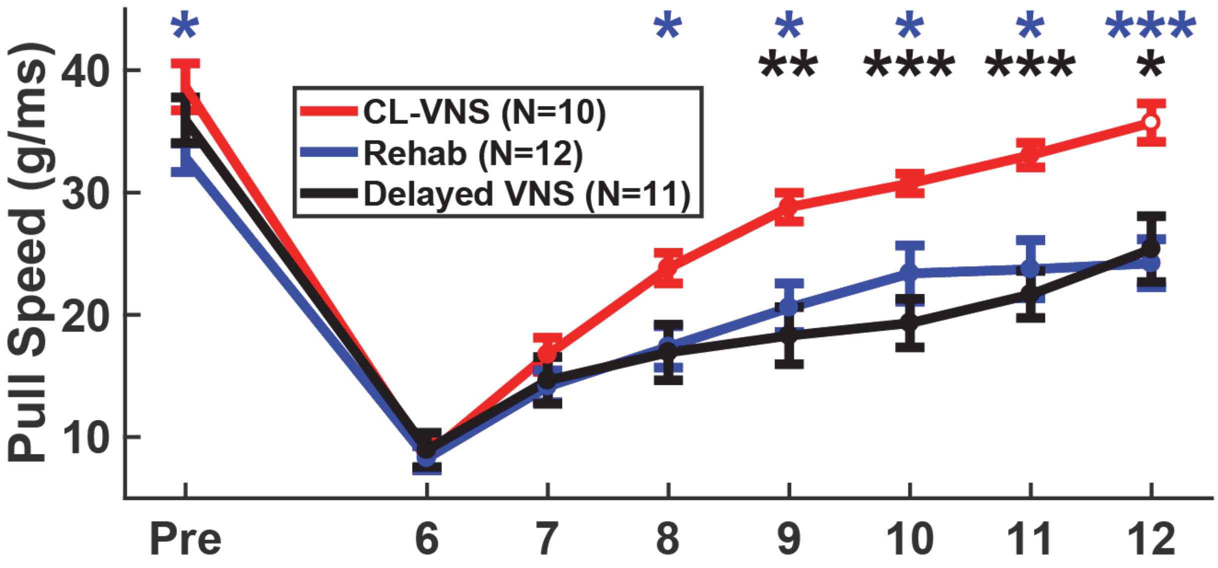

Supplementary Figure 6. CL-VNS improves recovery of speed of force generation. Peripheral nerve injury markedly reduced the speed of force generation. . Paired VNS significantly improved recovery of pull speed after peripheral nerve injury compared to both Rehab and Delayed VNS subjects (Two-way repeated measures ANOVA, main effect of group, $F[2,31]=6.23$, $p=0.005$). Asterisks indicate significant differences using t-tests across groups at each time point. The color of the asterisk denotes the group compared to CL-VNS. Error bars indicate S.E.M. *** $p < 0.001$, ** $p < 0.01$, * $p < 0.05$. Source data are provided as a Source Data file.

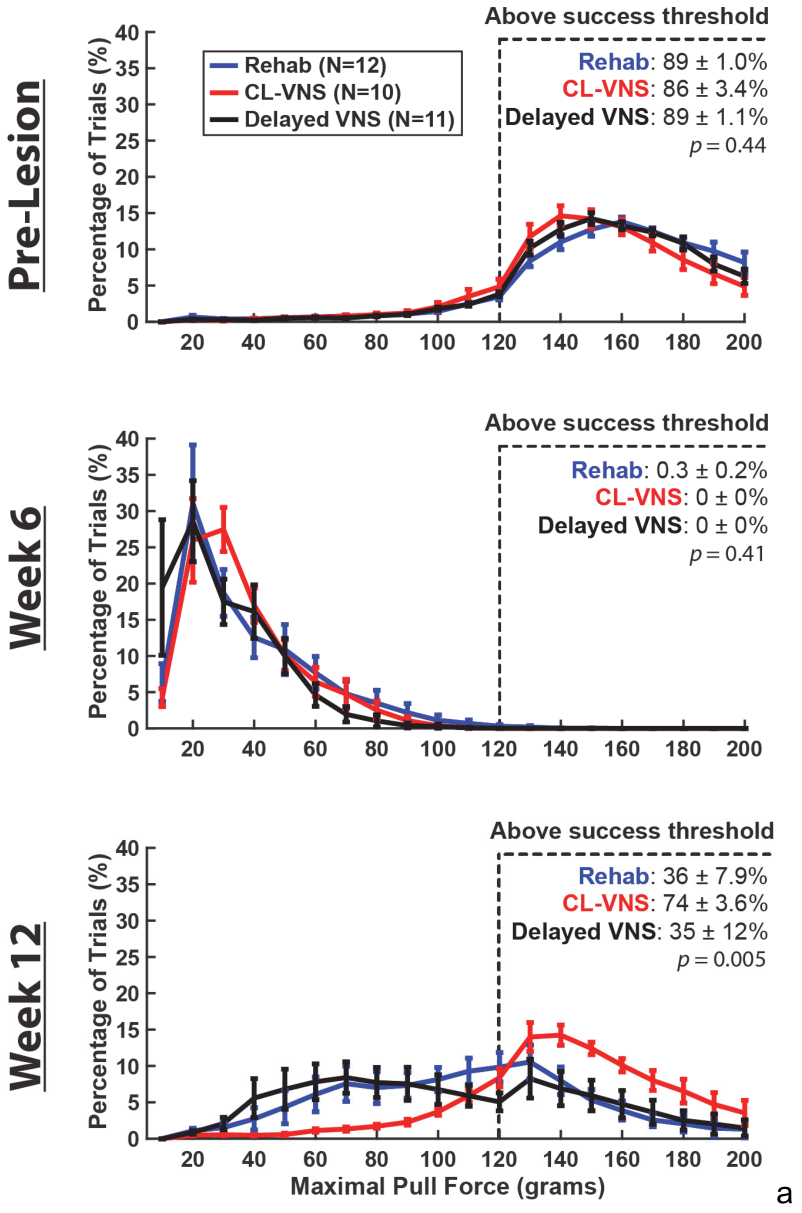

Supplementary Figure 7. Distribution of pull forces. Probability distribution histograms of maximal pull forces during Pre-Lesion training, the beginning of therapy (Week 6), and the end of therapy (Week 12). The numbers in the dashed box indicate the percentage of trials that exceed the 120-gram success threshold for each group. Note the rightward shift in the CL-VNS group following five weeks of VNS therapy compared to both rehabilitation alone (Rehab) and rehabilitation with delayed VNS (Delayed VNS). One-way ANOVA p-values are shown underneath the group averages. Error bars indicate S.E.M.

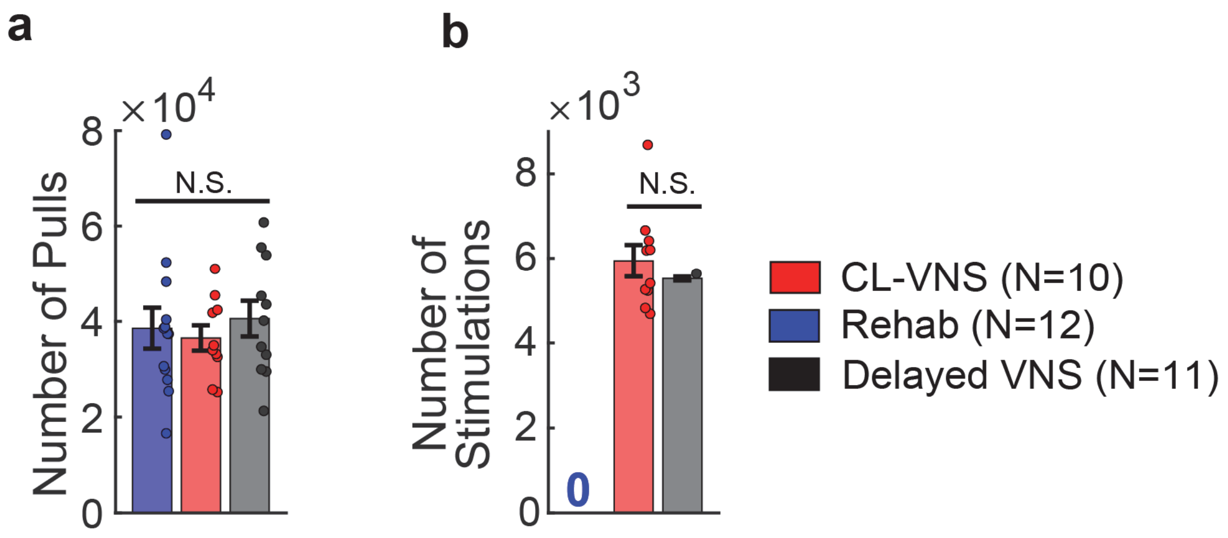

Supplementary Figure 8. Differences in the intensity of rehabilitative training or number of stimulations cannot account for recovery. (a) There were no differences in the total number of pull attempts performed during rehabilitative training across groups, demonstrating that the intensity of rehabilitation cannot explain the improved recovery in the CL-VNS group (One-way ANOVA, F[2,33]=0.27, p=0.77). (b) No difference was observed in the number of stimulations received by the CL-VNS and Delayed VNS groups, suggesting the amount of VNS cannot explain differences in recovery (Unpaired t-test, *p*=0.25). Circles depict individual subjects. Error bars indicate S.E.M. Source data are provided as a Source Data file.

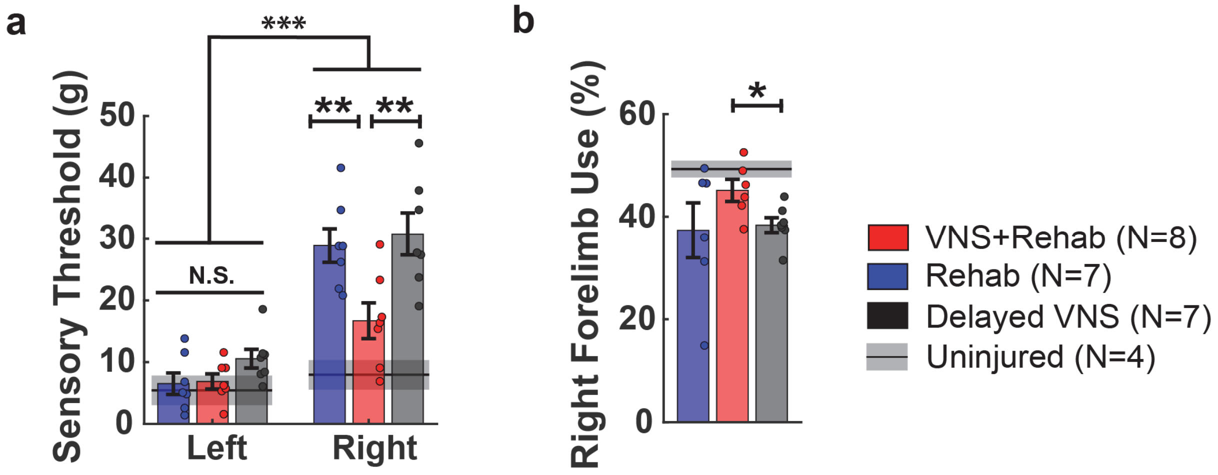

Supplementary Figure 9. Reversal of maladaptive plasticity restores sensory function after nerve injury. (a) Sensory thresholds in the injured paw were significantly increased, consistent with a loss of sensation following nerve injury (Two-Way ANOVA, main effect of paw, $F[1,41]=79.93$, $p=1.13\times10^{-10}$). CL-VNS significantly improved tactile sensation in the denervated forepaw (right) compared to both Rehab and Delayed VNS ($F[2,20]=6.35$, $p=0.008$). No change was observed in the uninjured forepaw (left) ($F[2,20]=2.22$, $p=0.14$). (b) CL-VNS improved spontaneous forelimb use during exploration on the cylinder task. Circles depict individual subjects. Error bars indicate S.E.M. *** $p < 0.001$, ** $p < 0.01$, * $p < 0.05$, N.S.: not significant. Source data are provided as a Source Data file.

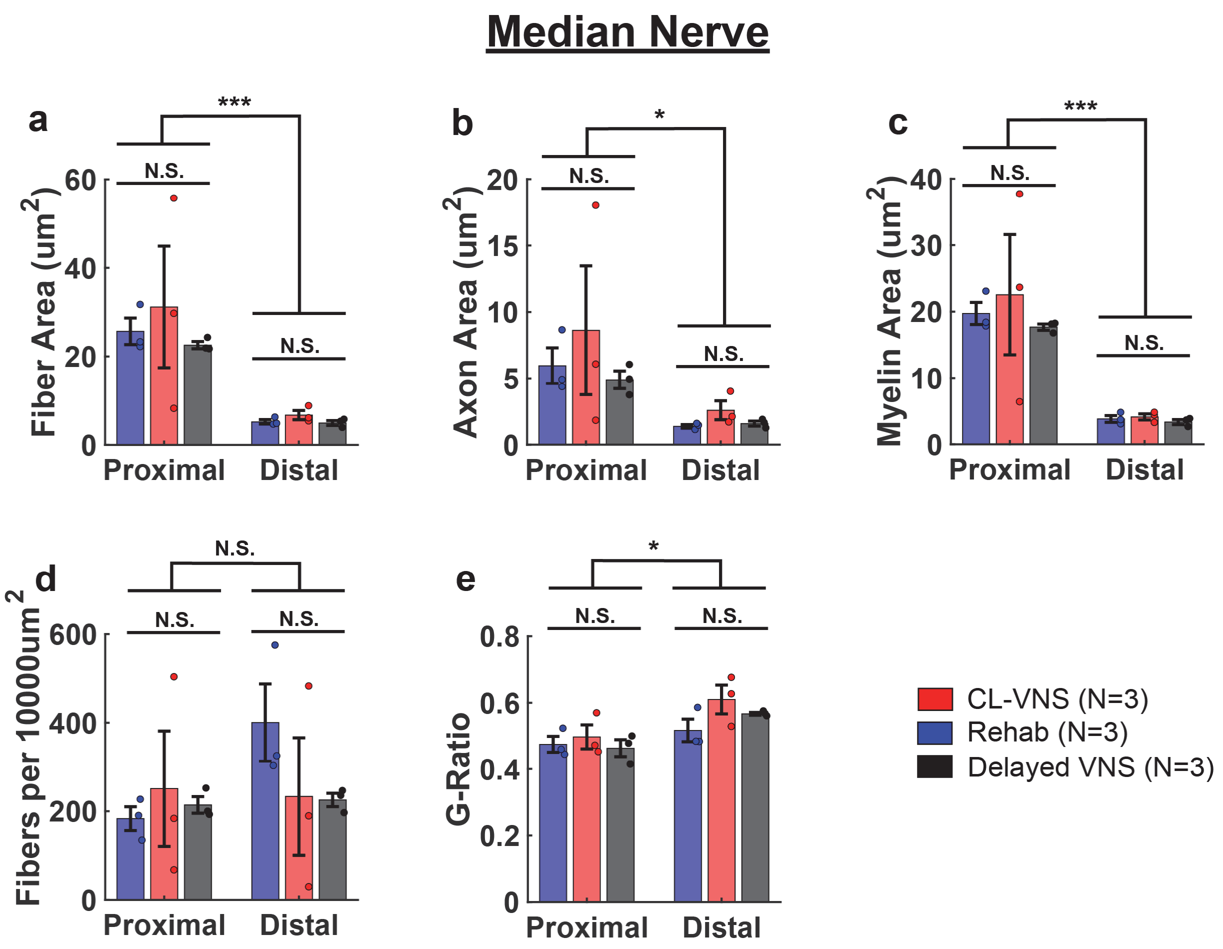

Supplementary Figure 10. Morphological analysis of injured median nerve. As expected, nerve injury reduced fiber area, axon area, and myelin area distal to the injury site. **(a)** No change was observed across groups for fiber area, **(b)** axon area, **(c)** myelin area, **(d)** fibers per 10000um^2, or **(e)** g-ratio, suggesting that VNS did not influence peripheral nerve architecture in the injured median nerve. Circles depict individual subjects. Error bars indicate S.E.M. *** $p < 0.001$, ** $p < 0.01$, * $p < 0.05$, N.S.: not significant. Statistics for each comparison are provided in Supplementary Table S15. Source data are provided as a Source Data file.

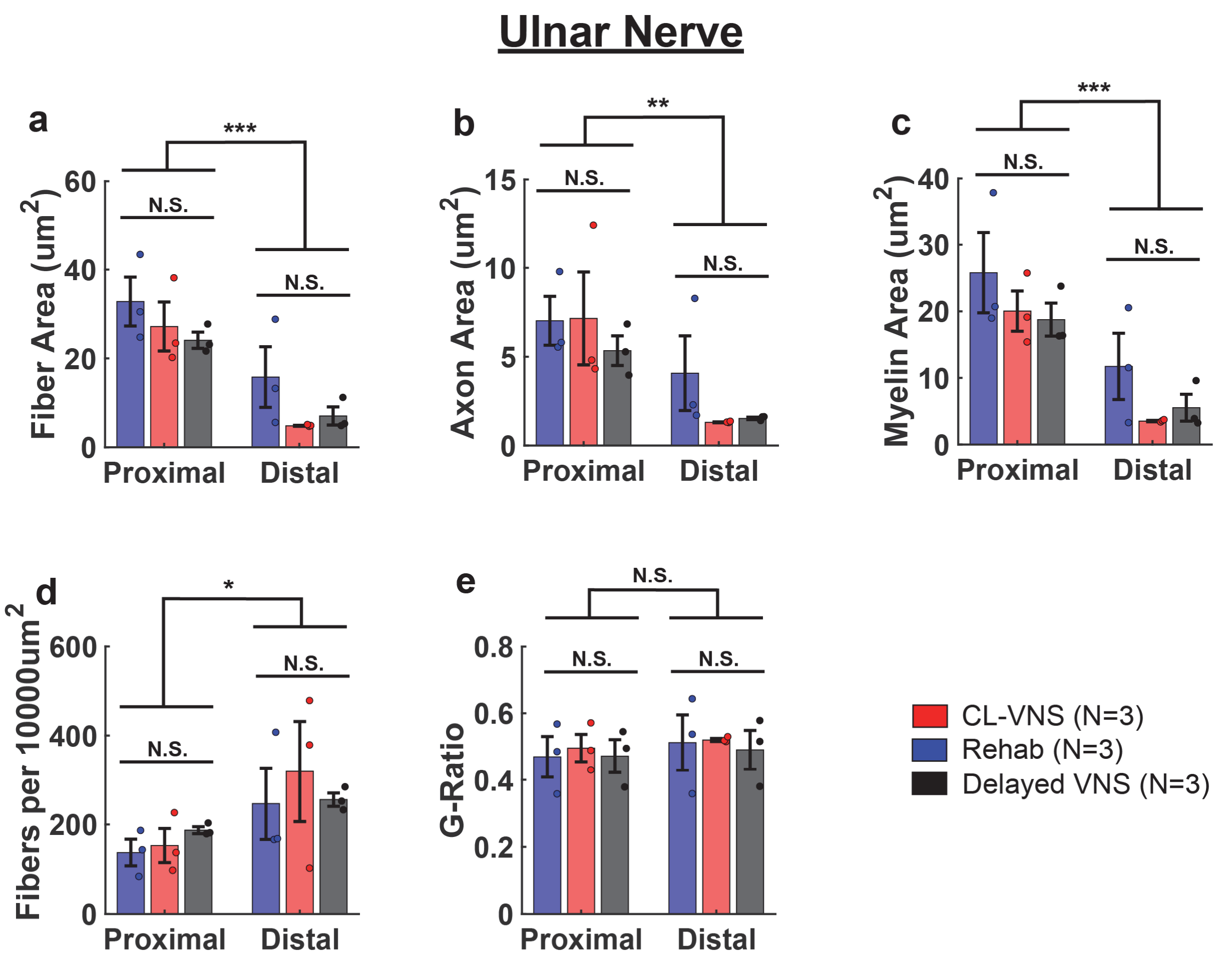

Supplementary Figure 11. Morphological analysis of injured ulnar nerve. (a) No change was observed across groups for fiber area, **(b)** axon area, **(c)** myelin area, **(d)** fibers per 10000um^2, or **(e)** g-ratio, suggesting that VNS did not influence peripheral nerve architecture in the injured ulnar nerve. Together with Fig. S8, these date indicate that VNS does not influence peripheral nerve health to enhance recovery. Circles depict individual subjects. Error bars indicate S.E.M. *** $p < 0.001$, ** $p < 0.01$, * $p < 0.05$, N.S.: not significant. Source data are provided as a Source Data file.

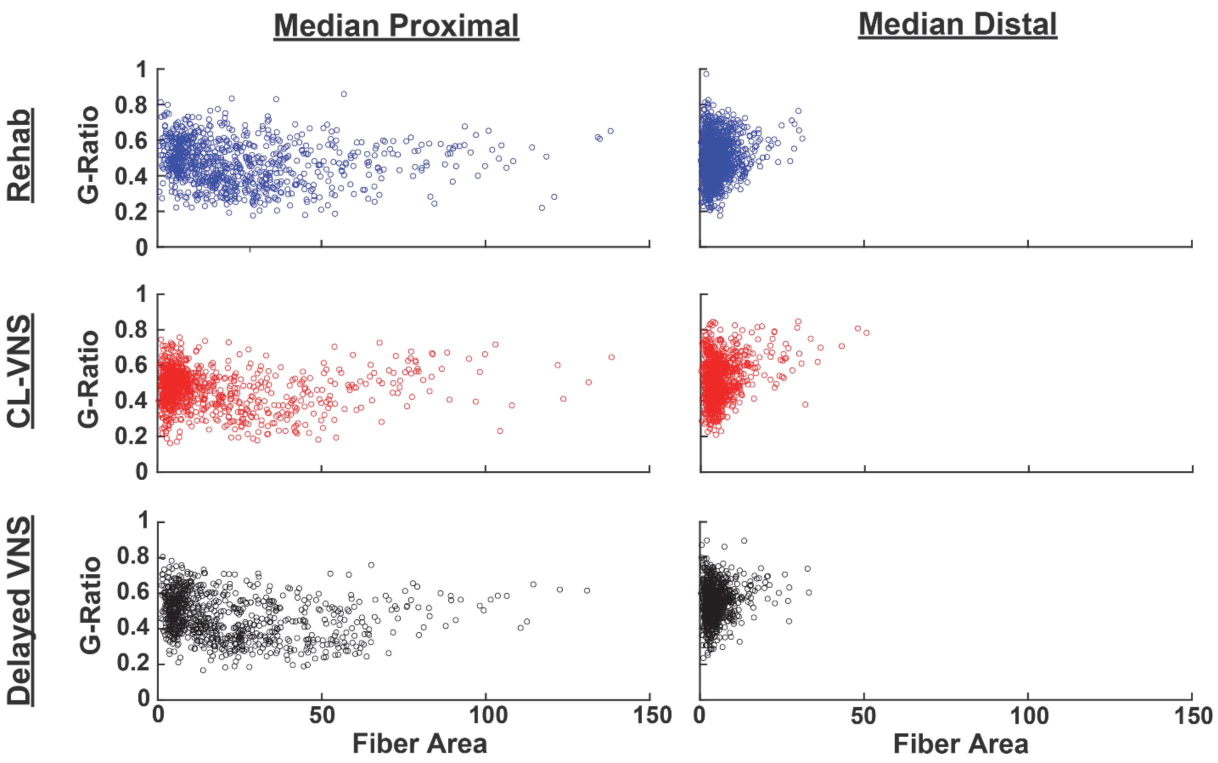

Supplementary Figure 12. Scatter plots of G-ratio as a function of fiber area in the median nerve both proximal and distal to injury site. Nerve injury substantially reduces fiber area without influencing G-ratio. No differences were observed across groups. Circles depict individual fibers.

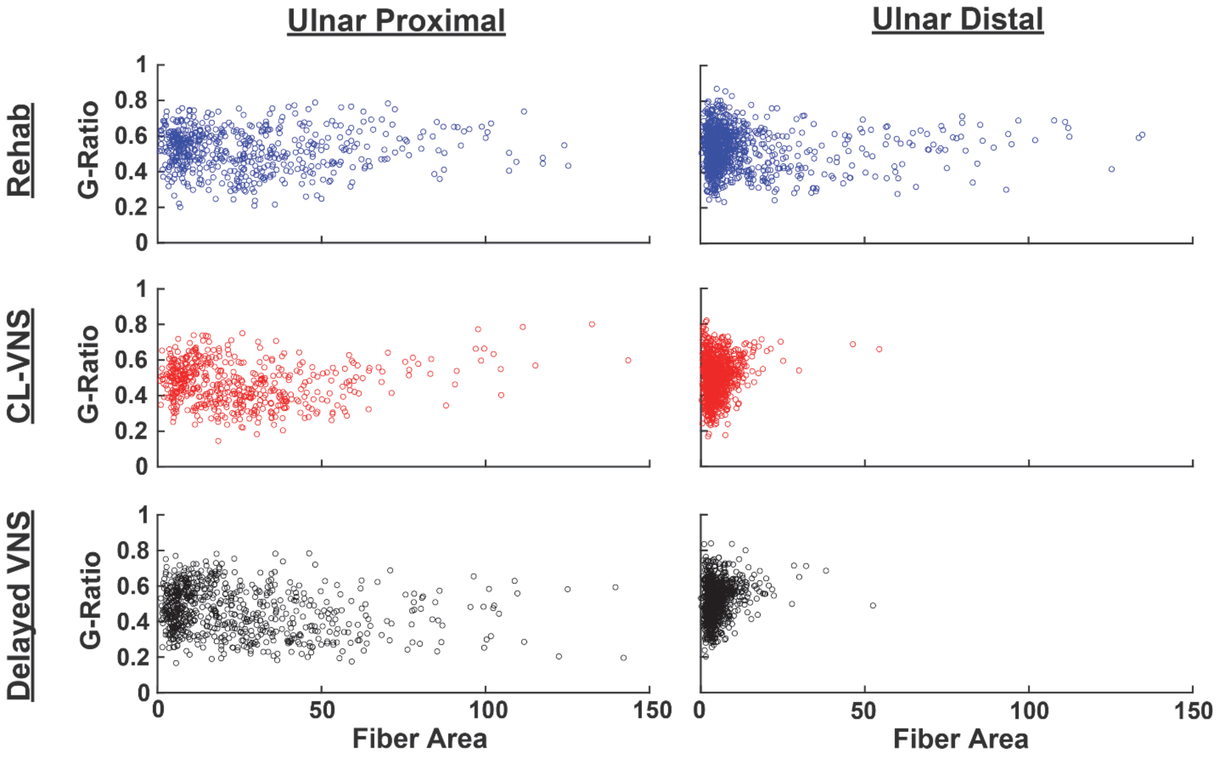

Supplementary Figure 13. Scatter plots of the G-ratio as a function of fiber area in the ulnar nerve both proximal and distal to injury site. Circles depict individual fibers.

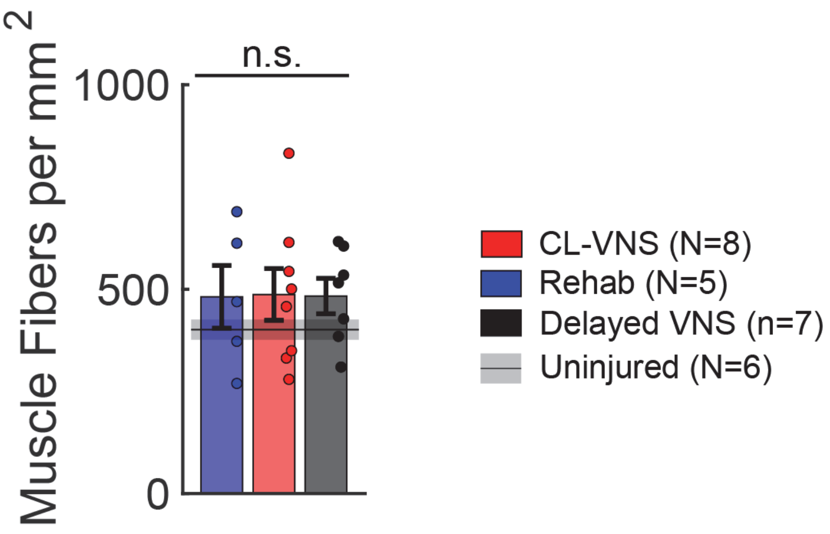

Supplementary Figure 14. Muscle fiber density in the flexor digitorum profundus (FDP) that was denervated by the nerve injury. No change in muscle fiber density of the denervated flexor digitorum profundus was observed across groups, suggesting that VNS does not improve muscle fiber density after nerve damage. Circles depict individual subjects. Error bars indicate S.E.M. *** $p < 0.001$, ** $p < 0.01$, * $p < 0.05$, N.S.: not significant. Source data are provided as a Source Data file.

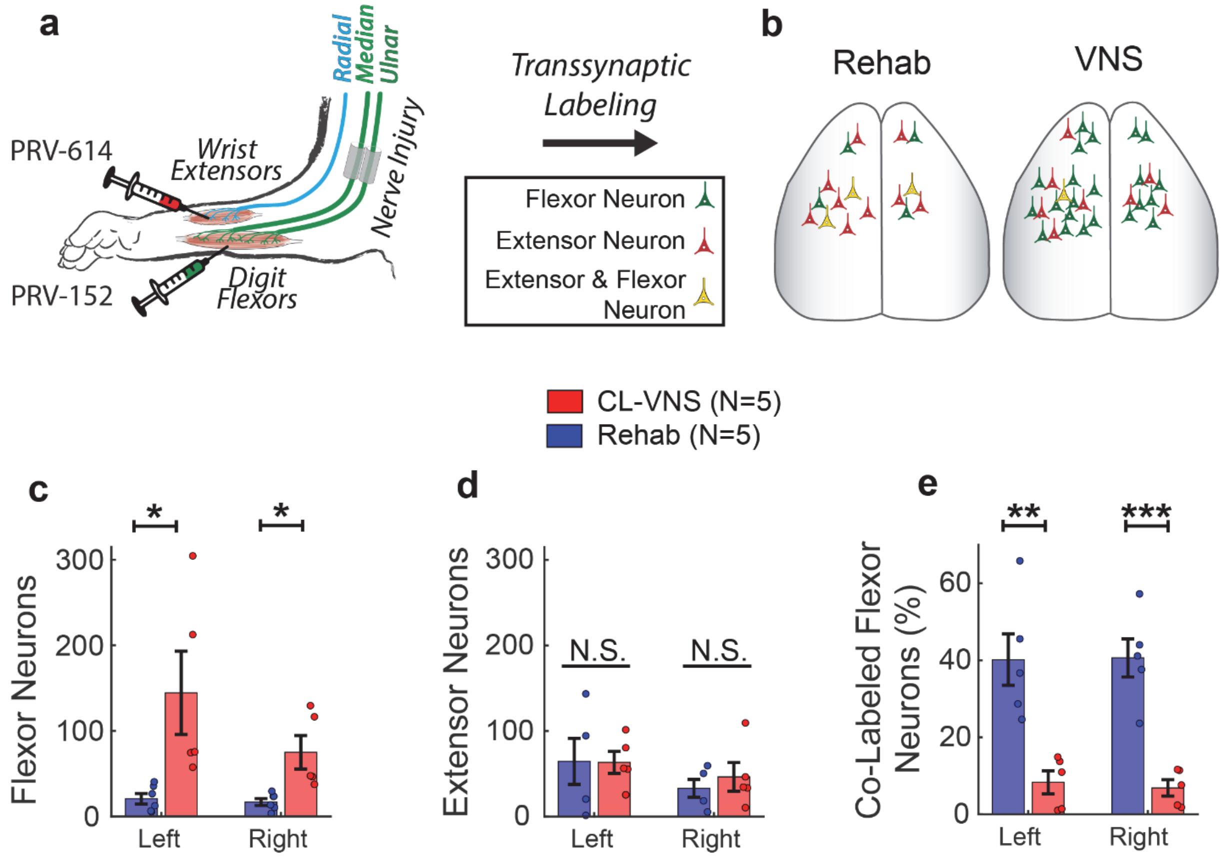

Supplementary Figure 15. VNS enhances synaptic reorganization in central networks after peripheral nerve damage. (a) Schematic detailing injection scheme of pseudorabies virus (PRV) tracing of synaptic connectivity. PRV-152, a retrograde transsynpatic tracer, was injected in to the grasping muscles to label synaptically connected networks with GFP. PRV-614 was injected in to the wrist extensors to label connected networks with RFP. **(b)** Schematic illustration detailing the top-down location of labeled PRV-positive neurons in the forebrain. GFP-positive neurons are depicted in green corresponding to flexor neurons, RFP-positive neurons depicted in red corresponding to extensor neurons, and double-labeled neurons depicted in yellow corresponding to cortical neurons connected to both flexor and extensor networks. **(c)** VNS paired with rehabilitation enhanced synaptic connectivity from cortical neurons in both the left and right hemispheres to digit flexors of the forelimb. **(d)** No changes were observed in either hemisphere of synaptic connectivity between cortical neurons and forelimb extensors. **(e)** VNS paired with rehabilitation significantly reduced the percentage of neurons displaying aberrant hyperconnectivity to both digit flexion and extensor networks in both hemispheres of motor cortex. Circles depict individual subjects. Error bars indicate S.E.M. *** $p < 0.001$, ** $p < 0.01$, * $p < 0.05$, N.S.: not significant. Source data are provided as a Source Data file.

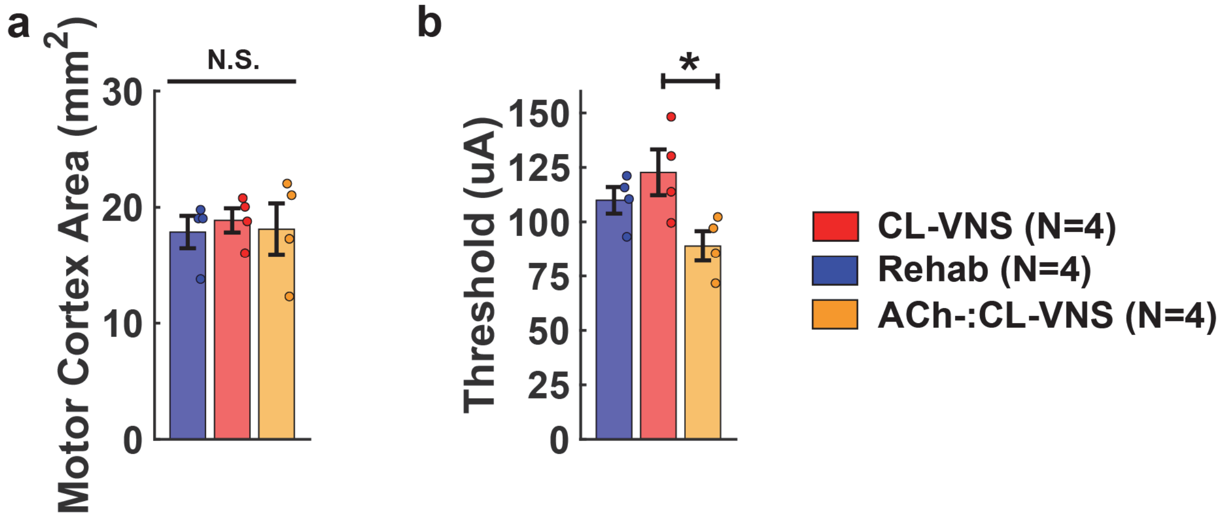

Supplementary Figure 16. Size and excitability of motor cortex. No difference was observed in (a) ICMS evoked motor cortex area (One-way ANOVA, $F[2,11]=0.1$, $p=0.90$). A slight but significant reduction was observed in (b) average threshold in the ACh-:CL-VNS subjects (One-way ANOVA, $F[2,11]=4.53$, $p=0.04$). Circles depict individual subjects. Error bars indicate S.E.M. *** $p < 0.001$, ** $p < 0.01$, * $p < 0.05$, N.S.: not significant. Source data are provided as a Source Data file.

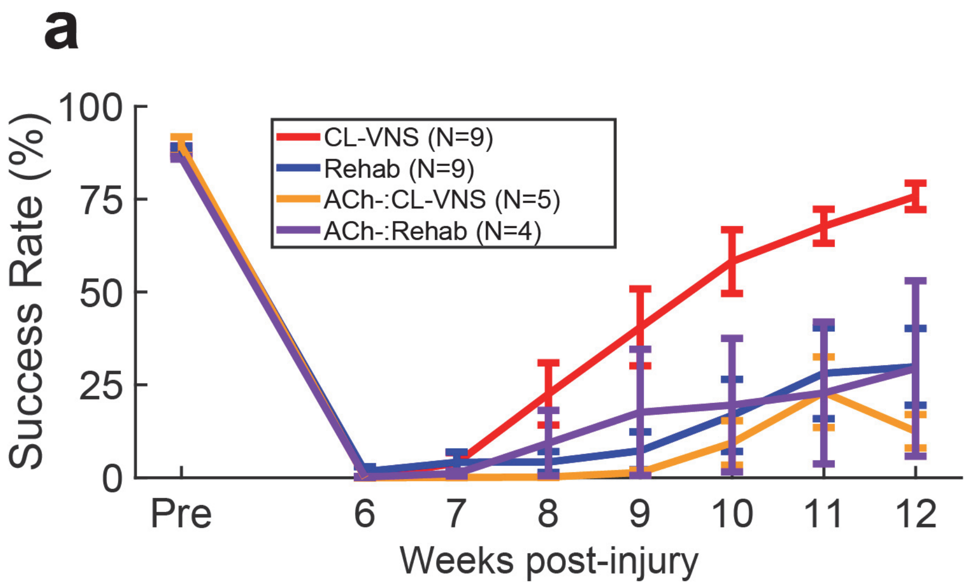

Supplementary Figure 17. Cortical cholinergic depletion does not prevent motor recovery during rehabilitation. No change was detected between Rehab alone (Rehab, N=9) and animals that received cortical cholinergic depletion and rehab only (ACh-:Rehab, N=4) for (a) success rate (Two-way ANOVA, F[1,10]=0.054 , p=0.83) or (b) pull force (Two-way ANOVA, F[1,10]=0.24 , p=0.63), demonstrating that cortical cholinergic depletion does not substantially prevent rehabilitation driven motor recovery after nerve damage. Error bars indicate S.E.M. *** p < 0.001, ** p < 0.01, * p < 0.05, N.S.: not significant. Source data are provided as a Source Data file.

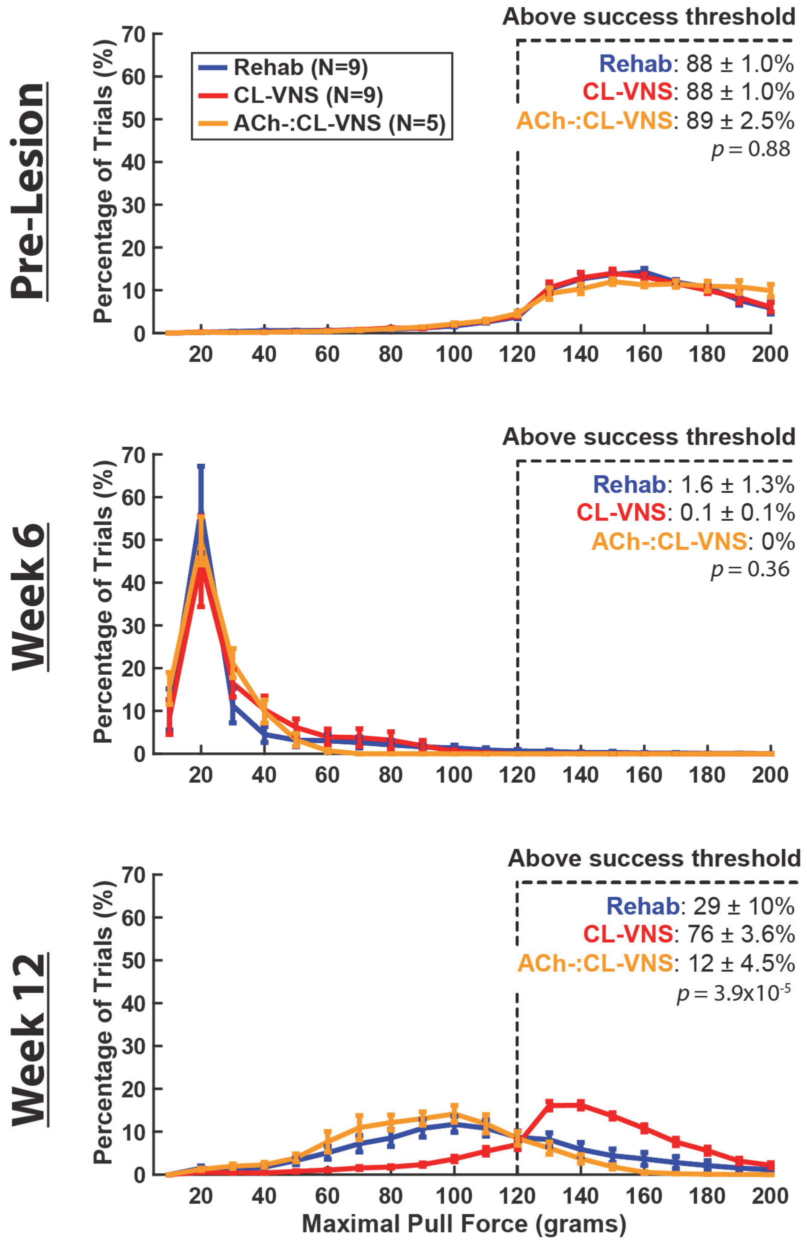

Supplementary Figure 18. Distribution of pull forces in Experiment 2. Probability distribution histograms of maximal pull forces during Pre-Lesion training, the beginning of therapy (Week 6), and the end of therapy (Week 12). The numbers in the dashed box indicate the percentage of trials that exceed the 120-gram success threshold for each group. Note the rightward shift in the CL-VNS group following five weeks of VNS therapy compared to both rehabilitation alone (Rehab) and ACh-depleted CL-VNS subjects (ACh-:CL-VNS). One-way ANOVA p-values are shown underneath the group averages. Error bars indicate S.E.M.

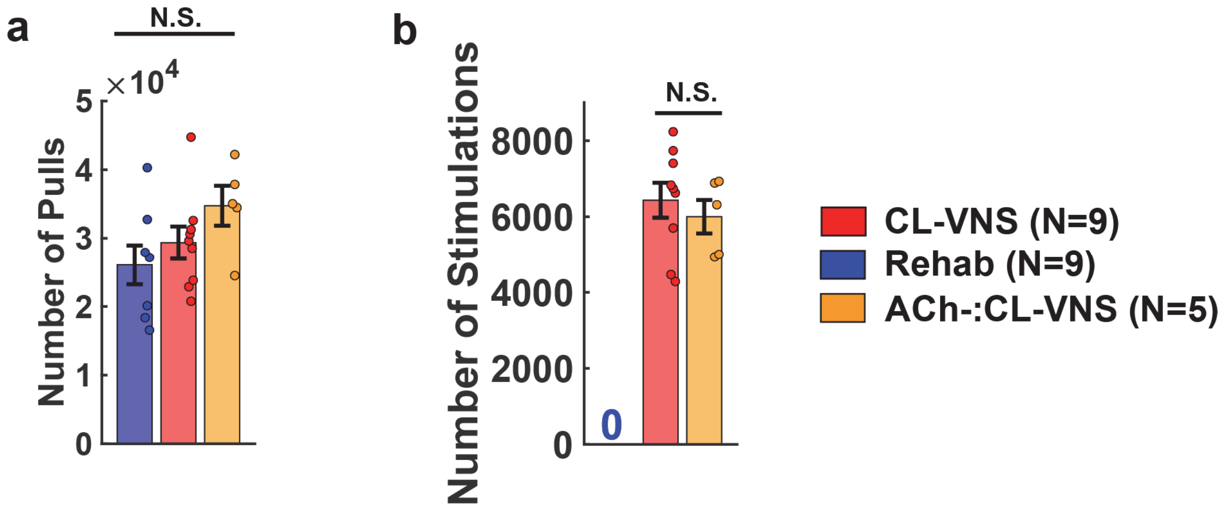

Supplementary Figure 19. Additional metrics of isometric pull task performance in the second set of experiments. (a) No differences in were observed across groups in the number of pull attempts performed during rehabilitative training, demonstrating that the intensity of rehabilitation cannot explain the improved recovery in CL-VNS subjects (One-way ANOVA ($F[2,22]=0.52$, $p=0.60$). **(b)** No significant difference was observed in the number of stimulations between Paired VNS and Delayed VNS subjects, suggesting the amount of stimulation cannot explain the improved recovery with CL-VNS (Unpaired t-test, p=0.38). Circles depict individual subjects. Error bars indicate S.E.M. *** $p < 0.001$, ** $p < 0.01$, * $p < 0.05$, N.S.: not significant. Source data are provided as a Source Data file.

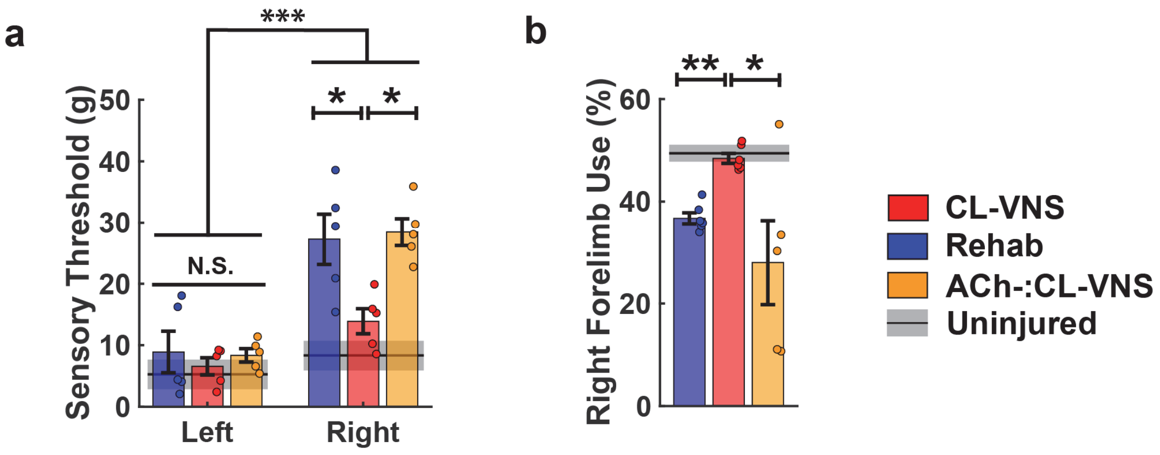

Supplementary Figure 20. Reversal of maladaptive plasticity is necessary to restore sensory function after nerve injury. (a) Sensory thresholds in the injured paw were significantly increased, consistent with a loss of sensation following nerve injury (Two-Way ANOVA, main effect of paw, $F[1,29]=51.7$, $p=1.97 \times 10^{-7}$). CL-VNS (N=5) significantly improved tactile sensation in the denervated forepaw (right) compared to both Rehab (N=5) and ACh-:CL-VNS (N=5) ($F[2,14]=7.54$, $p=0.008$). No change was observed in the uninjured forepaw (left) ($F[2,14]=0.31$, $p=0.74$). (b) CL-VNS (N=7) improved spontaneous forelimb use during exploration on the cylinder task, compared to CL-VNS after cholinergic depletion (ACh-:CL-VNS, N=5) or Rehab alone (N=6). Circles depict individual subjects. Error bars indicate S.E.M. *** $p < 0.001$, ** $p < 0.01$, * $p < 0.05$, N.S.: not significant. Source data are provided as a Source Data file.

II. Supplementary Tables

		Success Rate (%)							
ID	Group	Pre	Week 6	Week 7	Week 8	Week 9	Week 10	Week 11	Week 12
1	CL-VNS	88.86	0.00	10.43	44.27	53.38	70.82	76.38	55.46
2	CL-VNS	89.29	0.00	0.00	0.95	11.08	62.01	74.79	79.71
3	CL-VNS	85.62	0.00	3.83	39.02	58.14	68.90	78.80	94.79
4	CL-VNS	90.19	0.00	0.00	9.14	33.45	63.93	69.76	64.21
5	CL-VNS	87.12	0.23	0.00	20.89	65.35	56.85	83.31	76.75
6	CL-VNS	85.11	0.00	0.00	0.00	3.76	7.45	38.23	68.43
7	CL-VNS	92.27	0.00	0.19	29.26	58.74	53.49	68.19	76.13
8	CL-VNS	89.55	0.00	0.00	5.43	30.51	61.67	74.79	81.47
9	CL-VNS	81.23	0.00	3.02	6.61	19.18	20.28	40.57	63.00
10	CL-VNS	94.65	0.00	0.42	70.44	85.51	86.01	91.75	82.41
11	Rehab	91.91	0.00	0.28	6.82	39.97	40.67	35.87	37.37
12	Rehab	93.96	0.00	1.39	2.93	30.33	39.92	52.04	24.60
13	Rehab	93.08	0.00	1.56	9.67	19.27	10.75	23.88	23.88
14	Rehab	92.23	0.00	0.08	37.95	85.97	87.85	84.89	83.29
15	Rehab	92.15	0.00	0.00	15.44	58.34	38.18	39.35	71.62
16	Rehab	91.64	0.00	0.00	7.35	7.80	46.08	50.88	55.72
17	Rehab	85.21	0.53	35.64	20.43	53.39	48.58	68.67	67.72
18	Rehab	86.10	0.00	0.00	0.00	0.00	0.16	1.26	1.32
19	Rehab	88.39	0.00	13.68	54.90	56.66	63.17	44.00	23.97
20	Rehab	84.04	0.00	0.00	0.00	0.00	0.00	0.10	0.00
21	Rehab	86.81	0.00	0.00	0.00	0.00	3.27	1.22	18.09
22	Rehab	90.40	2.81	5.66	14.25	37.53	67.68	43.36	54.69
23	Rehab	83.87	0.00	0.00	0.00	0.00	0.08	1.07	0.84
24	Delayed VNS	86.80	0.10	0.00	0.12	14.16	6.74	21.93	25.88
25	Delayed VNS	93.16	0.00	0.00	0.11	17.73	53.60	78.89	76.02
26	Delayed VNS	94.20	0.00	0.12	0.06	0.67	0.96	1.31	2.69
27	Delayed VNS	84.67	0.00	0.00	0.30	10.89	52.08	84.94	80.31
28	Delayed VNS	87.36	0.00	0.00	0.04	0.00	0.08	11.24	26.95
29	Delayed VNS	87.74	0.00	0.00	0.00	0.00	0.00	0.00	0.00
30	Delayed VNS	88.34	0.00	3.62	49.35	88.29	88.85	93.71	93.88
31	Delayed VNS	88.99	0.17	6.10	5.69	5.55	4.37	1.29	1.10
32	Delayed VNS	88.57	0.00	0.00	1.67	0.05	0.09	2.40	1.68
33	Delayed VNS	94.42	0.00	0.07	2.60	11.28	5.48	61.05	83.53
34	Delayed VNS	84.58	0.00	0.00	0.00	0.00	0.00	0.00	0.00
68	Uninjured	92.67	84.27	78.10	90.82	91.27	91.80	90.32	93.48
69	Uninjured	91.66	92.75	88.87	91.92	93.86	91.85	89.82	92.63
70	Uninjured	88.44	73.73	83.33	90.37	89.19	91.40	91.96	91.53

71	Uninjured	85.01	80.83	87.28	90.40	88.15	92.00	81.11	92.44

Supplementary Table 1. Experiment 1: Success rates on isometric pull task for all animals during isometric pull training

		Pull Force (grams)							
ID	Group	Pre	Week 6	Week 7	Week 8	Week 9	Week 10	Week 11	Week 12
1.00	CL-VNS	138.24	45.48	89.72	116.59	121.55	131.47	132.92	122.38
2.00	CL-VNS	157.77	30.34	59.12	78.55	99.22	125.89	135.61	140.57
3.00	CL-VNS	156.03	38.04	78.97	110.39	127.75	140.98	151.73	179.84
4.00	CL-VNS	145.08	20.58	48.92	84.64	105.61	145.21	133.95	129.87
5.00	CL-VNS	158.31	23.40	43.87	97.06	125.41	126.10	139.74	142.22
6.00	CL-VNS	159.68	17.34	31.06	51.64	90.54	88.89	113.28	128.16
7.00	CL-VNS	148.68	34.75	64.29	106.86	124.66	122.49	133.79	138.13
8.00	CL-VNS	145.38	21.44	49.29	89.84	110.68	124.56	131.09	138.87
9.00	CL-VNS	144.21	40.94	85.01	89.21	100.58	96.79	111.94	128.35
10.00	CL-VNS	187.42	26.51	66.91	134.24	138.45	148.13	167.91	159.49
11.00	Rehab	158.68	42.55	73.30	92.25	112.45	114.11	112.04	114.11
12.00	Rehab	185.06	36.23	83.41	77.51	104.47	112.04	121.55	104.19
13.00	Rehab	176.27	42.32	74.16	92.41	102.89	89.30	96.68	98.62
14.00	Rehab	190.45	18.52	61.74	109.27	151.25	151.87	163.26	176.66
15.00	Rehab	159.96	17.08	35.33	94.35	126.57	112.21	112.98	131.42
16.00	Rehab	152.38	24.85	43.87	81.53	89.30	118.03	120.06	122.66
17.00	Rehab	143.25	61.98	109.22	98.82	122.23	118.32	132.63	132.12
18.00	Rehab	155.20	18.03	29.41	34.16	44.59	59.30	73.05	79.70
19.00	Rehab	151.25	40.31	78.02	122.49	125.97	135.09	112.94	101.64
20.00	Rehab	150.36	12.82	14.88	18.60	24.81	31.01	39.28	47.13
21.00	Rehab	163.78	23.68	25.20	33.74	50.20	71.64	68.50	90.47
22.00	Rehab	168.74	63.78	69.98	93.41	108.37	129.23	112.45	122.53
23.00	Rehab	153.11	15.55	34.31	48.72	52.23	54.74	70.82	67.30
24.00	Delayed VNS	147.54	19.41	49.70	70.88	93.53	94.41	89.70	98.23
25.00	Delayed VNS	165.05	26.05	45.06	72.76	102.12	121.96	141.81	130.65
26.00	Delayed VNS	162.12	15.30	31.42	43.00	54.57	73.18	73.59	83.10
27.00	Delayed VNS	149.18	19.84	47.13	57.46	83.08	120.44	138.73	136.74
28.00	Delayed VNS	142.61	24.49	38.09	38.67	54.74	66.80	92.96	96.58
29.00	Delayed VNS	148.62	36.14	36.59	37.51	32.02	39.79	46.20	48.03
30.00	Delayed VNS	149.42	26.12	80.36	119.03	145.15	144.14	156.45	171.26
31.00	Delayed VNS	169.65	34.65	74.83	75.34	57.76	52.74	57.76	77.35
32.00	Delayed VNS	160.80	4.57	43.45	40.25	41.17	51.69	62.21	76.39
33.00	Delayed VNS	169.53	38.42	54.41	73.68	85.70	79.81	127.87	145.10
34.00	Delayed VNS	143.91	8.62	14.06	13.15	20.58	27.44	42.08	47.57
68.00	Uninjured	164.70	157.77	151.05	163.83	161.23	163.40	164.70	169.90
69.00	Uninjured	147.32	200.68	174.23	173.82	172.91	155.57	170.70	166.07
70.00	Uninjured	147.84	136.39	142.91	150.32	162.60	163.45	161.33	161.33
71.00	Uninjured	158.98	154.56	168.95	161.33	150.32	150.32	143.97	168.11

Supplementary Table 2. Experiment 1: Pull forces on isometric pull task for all animals during isometric pull training.

ID	Group	Von Frey Left Paw (grams)	Von Frey Right Paw (grams)
1	CL-VNS	5.25	29
2	CL-VNS	11.5	17.25
3	CL-VNS	5.66	16.33
5	CL-VNS	9	23.25
6	CL-VNS	6.16	15.33
9	CL-VNS	1.5	9
10	CL-VNS	9	6.83
15	Rehab	6.75	20.75
16	Rehab	4.75	41.5
17	Rehab	5	34.66
18	Rehab	13.75	28.75
20	Rehab	11.5	28.75
21	Rehab	2.5	26.16
22	Rehab	1.33	21.83
28	Delayed VNS	11.16	23.66
29	Delayed VNS	8.33	27.33
30	Delayed VNS	10.66	45.5
31	Delayed VNS	11.33	37.83
32	Delayed VNS	18.5	19
33	Delayed VNS	8	34.67
34	Delayed VNS	6	27.67
68	Uninjured	5	5.16
69	Uninjured	5.6	11.16
70	Uninjured	7	9
71	Uninjured	11.3	6

Supplementary Table 3. Experiment 1: Von Frey sensory thresholds.

ID	Group	Forelimb Assymetry Index (%)
5	CL-VNS	43.75
3	CL-VNS	46.15
2	CL-VNS	37.5
7	CL-VNS	48.89
1	CL-VNS	42.11
8	CL-VNS	52.46
22	Rehab	46.51
20	Rehab	31.15
19	Rehab	14.81
16	Rehab	35.85
23	Rehab	49.35
21	Rehab	46.43
28	Delayed VNS	31.37
30	Delayed VNS	43.84
33	Delayed VNS	41.1
32	Delayed VNS	38.27
34	Delayed VNS	38.82
29	Delayed VNS	37.35
31	Delayed VNS	37.81
68	Uninjured	53.13
69	Uninjured	48.65
70	Uninjured	50

Supplementary Table 4. Experiment 1: Cylinder forelimb asymmetry index (%).

ID	Group	Myelinated Fibers per 100um^2		Fiber Area (um^2)		Myelin Area (um^2)		Axon Area (um^2)		G-Ratio	
		Proximal	Distal	Proximal	Distal	Proximal	Distal	Proximal	Distal	Proximal	Distal
21	Rehab	189.44	302.23	23.23	6.19	18.34	4.75	4.89	1.44	0.45	0.48
22	Rehab	133.88	574.43	31.65	4.78	23.02	3.66	8.63	1.11	0.52	0.48
23	Rehab	226.15	324.62	22.15	4.6	17.79	3.02	4.35	1.57	0.44	0.58
8	CL-VNS	503.04	188.68	8.17	5.33	6.36	3.24	1.81	2.08	0.47	0.62
9	CL-VNS	182.83	482.22	29.65	6.03	23.6	4.35	6.05	1.68	0.45	0.52
10	CL-VNS	66.9	28.82	55.66	8.76	37.66	4.76	17.99	3.99	0.56	0.67
27	Delayed VNS	199.16	234.76	24.22	5.24	18.19	3.6	6.02	1.64	0.49	0.55
28	Delayed VNS	251.45	245.75	21.82	5.7	18.08	3.82	3.73	1.88	0.41	0.57
29	Delayed VNS	192	195.88	21.66	3.85	16.73	2.61	4.92	1.23	0.47	0.56

Supplementary Table 5. Morphological analysis of median nerve.

ID	Group	Myelinated Fibers per 100um^2		Fiber Area (um^2)		Myelin Area (um^2)		Axon Area (um^2)		G-Ratio	
		Proximal	Distal	Proximal	Distal	Proximal	Distal	Proximal	Distal	Proximal	Distal
21	Rehab	82.98	406.49	24.69	13.16	18.91	11.48	5.78	1.68	0.48	0.35
22	Rehab	142.64	165.522	43.32	28.73	37.8	20.47	5.51	8.25	0.35	0.53
23	Rehab	185.87	167.53	30.41	5.5	20.64	3.23	9.76	2.27	0.56	0.64
8	CL-VNS	136.32	377.86	20.12	4.64	15.34	3.34	4.78	1.29	0.48	0.52
9	CL-VNS	225.61	477.7	23.36	5.03	19.06	3.68	4.29	1.34	0.42	0.51
10	CL-VNS	96.72	101.52	38.07	4.8	25.69	3.53	12.37	1.27	0.57	0.51
27	Delayed VNS	178.31	251.36	23.05	11.14	16.23	9.54	6.81	1.6	0.54	0.37
28	Delayed VNS	202.49	283.36	27.65	5.23	23.71	3.84	3.94	1.38	0.37	0.51
29	Delayed VNS	180.71	231.96	21.56	4.78	16.3	3.19	5.25	1.59	0.49	0.57

Supplementary Table 6. Morphological analysis of ulnar nerve.

ID	Group	Average Muscle Fiber Area	Average Muscle Fiber Density (fibers/mm^2)
6	CL-VNS	556	831
7	CL-VNS	997	330
8	CL-VNS	719	613
36	CL-VNS	930	349
35	CL-VNS	627	456
38	CL-VNS	1244	278
39	CL-VNS	866	542
10	CL-VNS	1144	499
18	Rehab	846	611
22	Rehab	817	688
46	Rehab	501	371
47	Rehab	1754	268
19	Rehab	910	468
25	Delayed VNS	822	426
26	Delayed VNS	727	615
28	Delayed VNS	983	514
23	Delayed VNS	707	383
30	Delayed VNS	827	308
31	Delayed VNS	889	604
32	Delayed VNS	875	533
62	Naive	860	370
63	Naive	920	356
64	Naive	1102	469
65	Naive	1067	445
66	Naive	1103	383
67	Naive	1180	376

Supplementary Table 7. Muscle fiber area and density.

ID	Group	Digit Flexion Area (mm^2)	Extension Area (mm^2)
1	CL-VNS	2.33	0.00
2	CL-VNS	2.00	0.38
3	CL-VNS	1.71	0.00
4	CL-VNS	4.38	0.00
5	CL-VNS	4.50	0.00
6	CL-VNS	2.38	0.00
8	CL-VNS	2.00	0.00
9	CL-VNS	3.25	0.00
11	Rehab	1.00	1.50
12	Rehab	0.63	0.38
13	Rehab	1.25	0.25
14	Rehab	0.13	1.33
15	Rehab	0.00	1.46
16	Rehab	2.88	0.75
17	Rehab	0.88	1.25
20	Rehab	2.13	0.50
21	Rehab	1.25	0.00
22	Rehab	2.50	0.38
23	Rehab	0.88	0.63
24	Delayed VNS	1.25	0.25
26	Delayed VNS	2.25	0.50
27	Delayed VNS	1.13	1.13
29	Delayed VNS	1.25	0.75
30	Delayed VNS	1.71	0.25
31	Delayed VNS	1.50	3.25
32	Delayed VNS	1.25	1.75
68	Uninjured	2.38	0.00
69	Uninjured	4.00	0.38
70	Uninjured	3.88	0.00
71	Uninjured	4.75	0.00

Supplementary Table 8. Experiment 1: Cortical area for intracortical microstimulation evoked movements.

ID	Group	GFP Left Hemisphere	GFP Right Hemisphere	GFP Total	RFP Left Hemisphere	RFP Right Hemisphere	RFP Total
35	CL-VNS	75	46	121	56	46	102
36	CL-VNS	212	116	328	55	34	89
38	CL-VNS	304	129	433	25	10	35
40	CL-VNS	74	37	111	80	33	113
42	CL-VNS	57	47	104	101	109	210
45	Rehab	26	23	49	20	18	38
48	Rehab	5	13	18	1	5	6
47	Rehab	40	23	63	143	59	202
51	Rehab	35	29	64	…	…	…
50	Rehab	12	10	22	…	…	…
52	Rehab	6	3	9	94	50	144
	□□denotes missing data due to technical issues.						

Supplementary Table 9. Cortical PRV-positive cell counts.

		Success Rate (%)							
ID	Group	Pre	Week 6	Week 7	Week 8	Week 9	Week 10	Week 11	Week 12
35.00	CL-VNS	86.81	0.00	8.18	25.48	40.77	75.14	48.61	81.37
36.00	CL-VNS	85.34	0.00	0.00	0.00	0.06	18.38	47.39	62.68
37.00	CL-VNS	89.97	0.83	25.15	66.85	73.40	87.50	87.03	92.34
38.00	CL-VNS	86.54	0.28	0.00	0.21	17.21	51.35	73.54	61.51
39.00	CL-VNS	91.73	0.00	0.00	44.23	78.69	78.49	71.90	77.59
40.00	CL-VNS	89.68	0.00	0.00	0.69	26.90	30.00	67.74	77.73
41.00	CL-VNS	85.12	0.00	1.66	46.83	73.71	68.68	70.05	69.78
42.00	CL-VNS	89.32	0.00	0.00	0.00	0.69	32.60	83.22	88.38
43.00	CL-VNS	91.55	0.00	0.44	18.68	52.87	81.76	59.72	70.34
44.00	Rehab	91.37	0.00	0.00	0.00	0.79	0.80	4.44	9.52
45.00	Rehab	91.08	0.00	0.00	0.00	12.32	80.65	89.49	88.83
46.00	Rehab	91.48	0.09	0.00	0.00	0.00	0.00	0.66	14.13
47.00	Rehab	88.69	2.29	1.48	0.59	0.17	1.46	9.78	11.60
48.00	Rehab	87.44	0.00	0.00	0.06	4.57	11.41	58.01	55.76
49.00	Rehab	89.45	0.00	0.13	0.00	0.00	0.00	0.00	2.27
50.00	Rehab	87.34	0.00	0.00	0.00	0.00	0.00	0.05	4.09
51.00	Rehab	84.27	11.97	23.28	14.69	0.64	4.91	11.11	20.77
52.00	Rehab	83.75	0.52	12.52	22.67	46.70	51.69	79.38	61.95
53.00	ACh-:CL-VNS	89.89	0.00	0.08	0.23	2.58	31.27	57.27	18.72
54.00	ACh-:CL-VNS	91.89	0.00	0.00	0.00	0.00	0.00	0.31	3.59
55.00	ACh-:CL-VNS	79.45	0.00	0.26	0.60	1.36	1.56	13.83	23.77
56.00	ACh-:CL-VNS	92.92	0.00	0.00	0.00	0.00	0.82	18.02	0.42
57.00	ACh-:CL-VNS	92.21	0.00	0.00	0.08	3.09	13.26	25.60	15.94

Supplementary Table 10. Experiment 2: Success rate on the isometric pull task for all animals during isometric pull training.

		Pull Force (grams)							
ID	Group	Pre	Week 6	Week 7	Week 8	Week 9	Week 10	Week 11	Week 12
35.00	CL-VNS	164.88	20.62	77.01	91.32	101.42	142.24	116.99	141.82
36.00	CL-VNS	162.28	11.34	31.98	51.34	66.91	100.16	118.67	126.25
37.00	CL-VNS	159.54	48.29	102.68	130.87	131.72	144.76	145.60	150.23
38.00	CL-VNS	152.08	14.64	41.94	70.82	94.92	121.20	129.19	126.67
39.00	CL-VNS	183.31	23.33	53.24	112.50	140.63	139.12	139.62	140.63
40.00	CL-VNS	149.54	14.32	29.06	64.85	107.17	103.59	126.33	132.64
41.00	CL-VNS	136.43	27.37	64.43	116.53	142.90	135.11	137.62	133.60
42.00	CL-VNS	149.46	15.57	21.60	32.85	62.32	108.64	139.80	141.07
43.00	CL-VNS	153.44	55.25	71.83	96.44	122.55	156.21	128.22	134.11
44.00	Rehab	182.22	19.67	28.82	52.60	73.64	72.27	78.67	90.57
45.00	Rehab	155.60	13.05	23.57	43.34	93.00	136.77	163.30	158.72
46.00	Rehab	173.65	13.56	18.08	24.61	39.17	52.74	78.35	98.94
47.00	Rehab	155.01	21.88	24.03	34.92	59.40	77.54	93.86	98.40
48.00	Rehab	141.25	10.04	17.58	46.21	77.35	94.92	124.56	123.55
49.00	Rehab	150.02	12.35	20.13	26.99	38.88	43.91	47.80	75.93
50.00	Rehab	143.50	13.56	21.10	36.67	39.18	49.22	61.28	77.85
51.00	Rehab	150.70	49.69	72.33	55.25	54.25	54.74	66.95	86.75
52.00	Rehab	151.62	44.21	86.32	95.59	117.49	121.28	142.75	127.17
53.00	ACh-:CL-VNS	173.19	14.31	26.09	55.13	61.02	101.00	124.98	91.32
54.00	ACh-:CL-VNS	171.97	13.05	32.82	45.87	53.74	54.24	74.33	83.87
55.00	ACh-:CL-VNS	138.16	13.89	17.25	23.57	45.45	71.33	95.95	98.05
56.00	ACh-:CL-VNS	178.86	24.61	37.67	49.72	59.77	70.82	71.82	64.29
57.00	ACh-:CL-VNS	193.20	20.20	26.93	41.66	69.86	86.69	107.31	101.00

Supplementary Table 11. Experiment 2: Pull force on the isometric pull task for all animals during isometric pull training.

Subject	Group	AChE fiber crossings	% Depleted
58	Control	212	--
59	Control	235	--
60	Control	210	--
61	Control	199	--
55	ACh-:VNS+Rehab	86	60%
53	ACh-:VNS+Rehab	6	97%
54	ACh-:VNS+Rehab	18.5	91%
57	ACh-:VNS+Rehab	49	77%
56	ACh-:VNS+Rehab	26	88%

Supplementary Table 12. Experiment 2: Percent of cortical cholinergic innervation depleted following lesions of the nucleus basalis.

ID	Group	Digit Flexion Area (mm^2)	Extension Area (mm^2)
5	VNS+Rehab	4.5	0
6	VNS+Rehab	2.37	0
8	VNS+Rehab	2	0
9	VNS+Rehab	3.25	0
20	Rehab	2.12	0.5
21	Rehab	1.25	0
22	Rehab	2.5	0.375
23	Rehab	0.875	0.625
53	ACh-:VNS+Rehab	0	1.75
54	ACh-:VNS+Rehab	0	3.625
56	ACh-:VNS+Rehab	0	3.25

Supplementary Table 13. Experiment 2: Cortical area for intracortical microstimulation evoked movements.

ID	Group	Von Frey Left Paw (grams)	Von Frey Right Paw (grams)
35	VNS+Rehab	2.33	15.83
36	VNS+Rehab	9	8.5
37	VNS+Rehab	9.17	15.17
39	VNS+Rehab	4.17	10.17
40	VNS+Rehab	8.17	19.83
45	Rehab	4	38.5
46	Rehab	4.33	20.83
47	Rehab	2	15.33
48	Rehab	18	29.33
50	Rehab	16.17	32.33
53	ACh-:VNS+Rehab	11.33	28
54	ACh-:VNS+Rehab	6.5	22.67
55	ACh-:VNS+Rehab	5.33	35.83
56	ACh-:VNS+Rehab	9.83	29.67

Supplementary Table 14. Experiment 2: Von Frey sensory thresholds

ID	Group	Forelimb Assymetry Index (%)
37	VNS+Rehab	48.04
35	VNS+Rehab	47
36	VNS+Rehab	46.53
40	VNS+Rehab	50.98
39	VNS+Rehab	51.72
41	VNS+Rehab	46.15
44	Rehab	35.09
47	Rehab	33.87
48	Rehab	38.3
49	Rehab	35.71
45	Rehab	36.11
50	Rehab	41.25
55	ACh-:VNS+Rehab	10.6
57	ACh-:VNS+Rehab	10.98
56	ACh-:VNS+Rehab	30.19
54	ACh-:VNS+Rehab	55

Supplementary Table 15. Experiment 2: Cylinder forelimb asymmetry index (%).

Median Nerve						
**Parameter**	**ANOVA**	**Factor**	**Segment**	**Degrees of Freedom**	**F-value**	**p-value**
Fibers per 100um^2	Two-way	Segment		[1,17]	1.02	0.33
Fibers per 100um^2	One-way		Proximal	[2,8]	0.19	0.83
Fibers per 100um^2	One-way		Distal	[2,8]	1.15	0.37
Fiber Area	Two-way	Segment		[1,17]	19.6	8.26×10^{-4}
Fiber Area	One-way		Proximal	[2,8]	0.28	0.76
Fiber Area	One-way		Distal	[2,8]	1.66	0.26
Myelin Area	Two-way	Segment		[1,17]	27.6	2.0×10^{-4}
Myelin Area	One-way		Proximal	[2,8]	0.21	0.82
Myelin Area	One-way		Distal	[2,8]	2.23	0.19
Axon Area	Two-way	Segment		[1,17]	7.37	0.018
Axon Area	One-way		Proximal	[2,8]	0.43	0.67
Axon Area	One-way		Distal	[2,8]	2.23	0.19
G-Ratio	Two-way	Segment		[1,17]	11.89	0.005
G-Ratio	One-way		Proximal	[2,8]	0.35	0.72
G-Ratio	One-way		Distal	[2,8]	2.12	0.20

Supplementary Table 16. Statistical values for the morphological analysis of median nerve.

Ulnar Nerve						
**Parameter**	**ANOVA**	**Factor**	**Segment**	**Degrees of Freedom**	**F-value**	**p-value**
Fibers per 100um^2	Two-way	Segment		[1,17]	5.45	0.37
Fibers per 100um^2	One-way		Proximal	[2,8]	0.82	0.49
Fibers per 100um^2	One-way		Distal	[2,8]	0.24	0.79
Fiber Area	Two-way	Segment		[1,17]	27.63	$2.02x10^{-4}$
Fiber Area	One-way		Proximal	[2,8]	0.91	0.45
Fiber Area	One-way		Distal	[2,8]	1.98	0.22
Myelin Area	Two-way	Segment		[1,17]	23.8	$3.79x10^{-4}$
Myelin Area	One-way		Proximal	[2,8]	0.82	0.49
Myelin Area	One-way		Distal	[2,8]	1.90	0.23
Axon Area	Two-way	Segment		[1,17]	11.49	0.005
Axon Area	One-way		Proximal	[2,8]	0.33	0.73
Axon Area	One-way		Distal	[2,8]	1.60	0.28
G-Ratio	Two-way	Segment		[1,17]	0.41	0.54
G-Ratio	One-way		Proximal	[2,8]	0.082	0.92
G-Ratio	One-way		Distal	[2,8]	0.07	0.93

Supplementary Table 17. Statistical values for the morphological analysis of ulnar nerve.

	Figure	Comparison	One-way ANOVA			Kruskal-Wallis Test		
			**D.O.F.**	**F value**	**p value**	**D.O.F.**	**Chi-Sq**	**p value**
**ICMS Digit Flexion**	Fig. 2a	Rehab vs. CL-VNS vs. Delayed VNS	2, 25	8.11	0.0022	2, 25	10.17	0.0062
**ICMS Extension**	Fig. 2a	Rehab vs. CL-VNS vs. Delayed VNS	2, 25	5.37	0.0122	2, 25	12.99	0.0015
**ICMS Multi-joint (%)**	Fig. 2c	Rehab vs. CL-VNS vs. Delayed VNS	2, 24	12.25	0.0003	2, 24	11.97	0.0025

	Figure	Comparison	Repeated Measures 2-way ANOVA		
			**D.O.F.**	F value	**p value**
**Success Rate**	Fig. 3a	Rehab vs. CL-VNS vs. Delayed VNS	14, 217	4.19	1.66x10-6
		Rehab vs. CL-VNS	7, 147	7.01	3.33x10-7
		CL-VNS vs. Delayed VNS	7, 133	5.85	6.21x10-6
		Rehab vs. Delayed VNS	7, 154	0.91	0.5

	Figure	Comparison	Repeated Measures 2-way ANOVA		
			**D.O.F.**	**F value**	**p value**
**Pull Force**	Fig. 2b	Rehab vs. CL-VNS vs. Delayed VNS	14, 217	3.96	4.68x10-6
		Rehab vs. CL-VNS	7, 147	5.76	6.58x10-6
		CL-VNS vs. Delayed VNS	7, 133	6.65	9.32x10-7
		Rehab vs. Delayed VNS	7. 154	0.81	0.58

	Figure	Comparison	One-way ANOVA			Kruskal-Wallis Test		
			**D.O.F.**	**F value**	**p value**	**D.O.F.**	**Chi-Sq**	**p value**
**Von Frey Right**	Fig. 3d	Rehab vs. CL-VNS vs. Delayed VNS	2, 20	6.35	0.0082	2, 20	7.715	0.021

	Figure	Comparison	One-way ANOVA			Kruskal-Wallis Test		
			**D.O.F.**	F value	**p value**	**D.O.F.**	Chi-Sq	**p value**
**Myelin Area - Proximal**	Fig. 4e	Rehab vs. CL-VNS vs. Delayed VNS	2, 8	0.21	0.81	2, 8	1.15	0.561

**Myelin Area - Distal**	Fig. 4e	Rehab vs. CL-VNS vs. Delayed VNS	2, 8	0.76	0.51	2, 8	1.42	0.491
**Muscle Fiber Area**	Fig. 4g	Rehab vs. CL-VNS vs. Delayed VNS	3, 25	0.81	0.5	3, 25	4.59	0.2

			**One-way ANOVA**			**Kruskal-Wallis Test**		
	**Figure**	**Comparison**	**D.O.F.**	**F value**	**p value**	**D.O.F.**	**Chi-Sq**	**p value**
**ICMS Digit Flexion**	Fig. 5a	Rehab vs. CL-VNS vs. ACh-:CL-VNS	2, 11	15.37	0.0013	2, 11	8.65	0.0132
**ICMS Extension**	Fig. 5a	Rehab vs. CL-VNS vs. ACh-:CL-VNS	2, 11	35.76	5.21x10-5	2, 11	9.43	0.009

			**Repeated Measures 2-way ANOVA**		
	**Figure**	**Comparison**	**D.O.F.**	F value	**p value**
**Success Rate**	Fig. 5c	Rehab vs. CL-VNS vs. ACh-:CL-VNS	14, 140	6.22	1.56x10-9
		Rehab vs. CL-VNS	7, 112	7.39	2.76x10-7
		CL-VNS vs. ACh-:CL-VNS	7, 84	11.53	3.53x10-10
		Rehab vs. ACh-:CL-VNS	7, 84	0.43	0.88

			**Repeated Measures 2-way ANOVA**		
	**Figure**	**Comparison**	**D.O.F.**	**F value**	**p value**
**Pull Force**	Fig. S15b	Rehab vs. CL-VNS vs. ACh-:CL-VNS	14, 140	4.13	5.23x10-6
		Rehab vs. CL-VNS	7, 112	4.64	1.38x10-4
		CL-VNS vs. ACh-:CL-VNS	7, 84	8.03	1.89x10-7
		Rehab vs. ACh-:CL-VNS	7, 84	0.81	0.58

			**One-way ANOVA**			**Kruskal-Wallis Test**		
	**Figure**	**Comparison**	**D.O.F.**	**F value**	**p value**	**D.O.F.**	**Chi-Sq**	**p value**
**Von Frey Right**	Fig. 5d	Rehab vs. CL-VNS vs. ACh-:CL-VNS	2, 14	7.54	0.0076	2, 14	7.98	0.0185

Supplementary Table 18. ANOVAs and Kruskal-Wallis tests for all figures.

	Figure	Rehab Mean	Rehab CI	Rehab STD	VNS Mean	VNS CI	VNS STD	Delayed VNS Mean	Delayed VNS CI	Delayed VNS STD
**ICMS Digit Flexion**	Fig. 2a	1.227	0.62	0.923	2.817	0.918	1.098	1.476	0.364	0.393
**ICMS Extension**	Fig. 2a	0.765	0.357	0.531	0.0469	0.11	0.132	1.125	0.996	1.077
**ICMS Multi-Joint (%)**	Fig. 2c	51.56	16.63	23.25	9.91	12.14	14.52	70.47	31.4	33.96

**Success Rate**	Figure	Rehab Mean	Rehab Confidence Interval	Rehab STD	VNS Mean	VNS Confidence Interval	VNS STD	Delayed VNS mean	Delayed VNS Confidence Interval	Delayed VNS STD
**Pre**	Fig.3a	0.8922	0.0219	0.0362	0.8861	0.0244	0.034	0.8898	0.0234	0.0349
**Week 6**		0.0026	0.0047	0.0078	0.0002	0.0005	0.0007	0.0002	0.0004	0.0006
**Week 7**		0.0448	0.0612	0.1013	0.0179	0.0239	0.0335	0.009	0.0137	0.0204
**Week 8**		0.1306	0.1002	0.1658	0.226	0.165	0.2306	0.0545	0.0985	0.1467
**Week 9**		0.2994	0.1705	0.2822	0.4191	0.1886	0.2636	0.1351	0.1723	0.2565
**Week 10**		0.3434	0.1768	0.2926	0.5514	0.1695	0.2369	0.1929	0.2068	0.3079
**Week 11**		0.3435	0.1664	0.2754	0.6966	0.1237	0.1729	0.3243	0.2601	0.3872
**Week 12**		0.3562	0.172	0.2847	0.7423	0.0824	0.1152	0.3564	0.2641	0.3931

**Pull Force**	Figure	Rehab Mean	Rehab Confidence Interval	Rehab STD	VNS Mean	VNS Confidence Interval	VNS STD	Delayed VNS mean	Delayed VNS Confidence Interval	Delayed VNS STD
**Pre**	Fig.3b	162.1918	8.5968	14.2263	154.0783	9.8683	13.7949	155.3128	6.876	10.2351
**Week 6**		32.1305	10.4255	17.2524	29.8817	6.8469	9.5713	23.0549	7.347	10.9362
**Week 7**		56.3715	16.8362	27.8609	61.7149	13.574	18.9751	46.8288	12.5352	18.6588
**Week 8**		76.712	19.5537	32.358	95.9016	16.3434	22.8464	58.34	19.0009	28.2832
**Week 9**		93.4872	23.2761	38.5179	114.4445	10.969	15.3336	70.0379	24.2097	36.0366
**Week 10**		99.7608	21.5866	35.7221	125.051	13.7993	19.2901	79.3094	25.0087	37.2259
**Week 11**		102.7876	19.687	32.5787	135.1943	11.7086	16.3675	93.5775	27.758	41.3185
**Week 12**		106.8108	19.8148	32.79	140.7887	12.2987	17.1924	101.0001	27.0498	40.2641

	Figure	Rehab Mean	Rehab CI	Rehab STD	VNS Mean	VNS CI	VNS STD	Delayed VNS Mean	Delayed VNS CI	Delayed VNS STD
**Von Frey Right**	Fig. 3d	28.91	6.71	7.25	16.71	7.096	7.67	30.8	8.37	9.06

	Figure	Rehab Mean	Rehab CI	Rehab STD	VNS Mean	VNS CI	VNS STD	Delayed VNS Mean	Delayed VNS CI	Delayed VNS STD
**PRV GFP Total**	Fig. 4e	37.5	25.34	24.15	219.4	188.48	151.79	N/A	N/A	N/A
**PRV RFP Total**	Fig. 4f	97.5	145.24	91.27	109.8	78.86	63.51	N/A	N/A	N/A
**PRV Double Labeled (%)**	Fig. 4g	36.69	17.26	10.84	7.74	7.4	5.96	N/A	N/A	N/A
**Myelin Area - Proximal**	Fig. 4d	19.72	7.14	2.87	22.54	38.94	15.6779	17.67	2.02	0.81
**Myelin Area - Distal**	Fig. 4d	3.81	2.16	0.871	4.122	1.96	0.789	3.348	1.597	0.6429
**Muscle Fiber Area**	Fig. 4b	965.6	581.36	468.21	885.37	203.13	242.97	832.85	88.28	95.45

	Figure	Rehab Mean	Rehab CI	Rehab STD	VNS Mean	VNS CI	VNS STD	ACh:-/CL-VNS mean	ACh:-/CL-VNS Confidence Interval	ACh:-/CL-VNS STD
**ICMS Digit Flexion**	Fig. 5a	1.687	1.198	0.753	3.031	1.767	1.11	0	0	0
**ICMS Extension**	Fig. 5a	0.375	0.429	0.27	0	0	0	2.781	1.323	0.831

**<u>Success Rate</u>**		Rehab Mean	Rehab Confidence Interval	Rehab STD	VNS Mean	VNS Confidence Interval	VNS STD	ACh:-/CL-VNS mean	ACh:-/CL-VNS Confidence Interval	ACh:-/CL-VNS STD
**Pre**	Fig.5c	0.8832	0.0223	0.029	0.8845	0.0196	0.0255	0.8927	0.0696	0.056
**Week 6**		0.0165	0.0303	0.0394	0.0012	0.0022	0.0028	0	0	0

**Week 7**		0.0416	0.0635	0.0826	0.0394	0.0645	0.0839	0.0007	0.0014	0.0011
**Week 8**		0.0422	0.0649	0.0844	0.2255	0.1929	0.251	0.0018	0.0031	0.0025
**Week 9**		0.0724	0.1179	0.1534	0.4048	0.2392	0.3111	0.0141	0.0178	0.0143
**Week 10**		0.1677	0.2242	0.2916	0.5821	0.198	0.2575	0.0938	0.1662	0.1339
**Week 11**		0.281	0.2825	0.3676	0.6769	0.1057	0.1375	0.23	0.2638	0.2124
**Week 12**		0.2988	0.2382	0.3099	0.7575	0.0821	0.1068	0.1249	0.1246	0.1003

**Pull Force**		**Rehab Mean**	**Rehab Confidence Interval**	**Rehab STD**	**VNS Mean**	**VNS Confidence Interval**	**VNS STD**	**ACh:-/CL-VNS mean**	**ACh:-/CL-VNS Confidence Interval**	**ACh:-/CL-VNS STD**
**Pre**		155.9515	10.3734	13.4953	156.7739	10.0237	13.0403	171.0751	25.1353	20.2432
**Week 6**		22.002	11.285	14.6812	25.6358	12.0887	15.7268	17.2099	6.2239	5.0126
**Week 7**		34.6609	19.8222	25.7878	54.8615	20.3769	26.5094	28.1538	9.5555	7.6957
**Week 8**	Fig.S15b	46.2411	16.3596	21.2831	85.2788	25.0338	32.5677	43.1892	14.9489	12.0394
**Week 9**		65.8171	20.9151	27.2096	107.8376	22.8126	29.6781	57.9661	11.252	9.0621
**Week 10**		78.15	25.5153	33.1942	127.8913	15.4827	20.1423	76.8146	22.0186	17.7332
**Week 11**		95.28	30.4014	39.5507	131.3395	7.6341	9.9317	94.8784	27.8738	22.4488
**Week 12**		104.2088	20.91	27.2028	136.3348	5.9869	7.7887	87.7055	18.2046	14.6614

	**Figure**	**Rehab Mean**	**Rehab CI**	**Rehab STD**	**VNS Mean**	**VNS CI**	**VNS STD**	**ACh:-/CL-VNS mean**	**ACh:-/CL-VNS Confidence Interval**	**ACh:-/CL-VNS STD**
**Von Frey Right**	Fig. 5e	27.26	11.44	9.21	13.9	5.675	4.571	28.434	6.069	4.887

Supplementary Table 19. Means and variance for all figures.

	Student's t-test p-value			Effect Size (Cohen's d)			Wilcoxon rank-sum p-value			Lilliefors Test p-value		
	CL-VNS vs. Rehab	CL-VNS vs. Delayed VNS	Rehab vs. Delayed VNS	CL-VNS vs. Rehab	CL-VNS vs. Delayed VNS	Rehab vs. Delayed VNS	CL-VNS vs. Rehab	CL-VNS vs. Delayed VNS	Rehab vs. Delayed VNS	CL-VNS	Delayed VNS	Rehab
**ICMS Digit Flexion**	0.0032	0.0092	0.512	1.57	1.798	0.377	0.0084	0.0018	0.23	0.061	0.079	0.152
**ICMS Extension**	0.0017	0.014	0.35	2.16	1.78	0.447	0.00087	0.00093	0.705	0.001	0.422	0.368
**ICMS Multi-Joint (%)**	0.00043	0.00049	0.191	2.205	2.4981	0.661	0.00077	0.011	0.10078	0.0096	0.038	0.271

	Student's t-test p-value			Effect Size (Cohen's d)			Wilcoxon rank-sum p-value			Lilliefors Test p-value		
**<u>Success Rate</u>**	CL-VNS vs. Rehab	CL-VNS vs. Delayed VNS	Rehab vs. Delayed VNS	CL-VNS vs. Rehab	CL-VNS vs. Delayed VNS	Rehab vs. Delayed VNS	CL-VNS vs. Rehab	CL-VNS vs. Delayed VNS	Rehab vs. Delayed VNS	CL-VNS	Delayed VNS	Rehab
**Pre**	0.6861	0.8047	0.876	0.1732	0.1097	0.0648	0.6869	0.9159	0.9538	0.5	0.1147	0.116
**Week 6**	0.3578	0.9508	0.3369	0.5497	0.0273	0.5556	0.6718	0.7286	1	0.001	0.001	0.001
**Week 7**	0.4303	0.4664	0.2626	0.3999	0.3302	0.589	0.8182	0.4352	0.3123	0.001	0.001	0.001
**Week 8**	0.2605	0.054	0.2508	0.4815	0.9091	0.4868	0.3038	0.0219	0.119	0.1825	0.001	0.0965
**Week 9**	0.3116	0.0217	0.1528	0.4385	1.0921	0.6101	0.2501	0.0101	0.2408	0.5	0.001	0.3804
**Week 10**	0.0814	0.0079	0.2334	0.7857	1.316	0.501	0.0586	0.0054	0.2706	0.0349	0.001	0.3292
**Week 11**	0.0019	0.0116	0.8886	1.5749	1.3292	0.058	0.007	0.0725	0.9077	0.0433	0.0468	0.2019
**Week 12**	0.0006	0.0076	0.9992	1.9309	1.5185	0.0004	0.0032	0.0725	0.9538	0.5	0.0464	0.2267

	Student's t-test p-value			Effect Size (Cohen's d)			Wilcoxon rank-sum p-value			Lilliefors Test p-value		
**<u>Pull Force</u>**	CL-VNS vs. Rehab	CL-VNS vs. Delayed VNS	Rehab vs. Delayed VNS	CL-VNS vs. Rehab	CL-VNS vs. Delayed VNS	Rehab vs. Delayed VNS	CL-VNS vs. Rehab	CL-VNS vs. Delayed VNS	Rehab vs. Delayed VNS	CL-VNS	Delayed VNS	Rehab
**Pre**	0.1841	0.8172	0.1953	0.5791	0.1028	0.5624	0.145	0.5035	0.2237	0.0958	0.0331	0.3059
**Week 6**	0.7154	0.1462	0.1466	0.1677	0.6658	0.6439	0.9259	0.1697	0.297	0.5	0.5	0.1525
**Week 7**	0.6088	0.0859	0.3446	0.2282	0.7911	0.4103	0.535	0.098	0.643	0.5	0.5	0.4694

**Week 8**	0.1265	0.0036	0.1566	0.6952	1.4693	0.6059	0.2265	0.0043	0.1178	0.5	0.3672	0.0739
**Week 9**	0.1206	0.0019	0.1403	0.7783	1.7289	0.6291	0.2778	0.0043	0.1178	0.478	0.4014	0.1097
**Week 10**	0.0567	0.0025	0.1842	0.9194	1.6187	0.5607	0.0586	0.0035	0.2466	0.0821	0.5	0.0257
**Week 11**	0.0092	0.0078	0.5476	1.3242	1.4429	0.2493	0.0058	0.0448	0.643	0.2984	0.5	0.0631
**Week 12**	0.0073	0.0094	0.7004	1.3596	1.385	0.1591	0.0048	0.0378	0.643	0.0423	0.5	0.5

	**Student's t-test p-value**			**Effect Size (Cohen's d)**			**Wilcoxon rank-sum p-value**			**Lilliefors Test p-value**		
	**CL-VNS vs. Rehab**	**CL-VNS vs. Delayed VNS**	**Rehab vs. Delayed VNS**	**CL-VNS vs. Rehab**	**CL-VNS vs. Delayed VNS**	**Rehab vs. Delayed VNS**	**CL-VNS vs. Rehab**	**CL-VNS vs. Delayed VNS**	**Rehab vs. Delayed VNS**	**CL-VNS**	**Delayed VNS**	**Rehab**
**Von Frey Right**	0.0099	0.0085	0.673	1.63	1.68	0.23	0.023	0.011	0.779	0.5	0.49	0.35

	**Student's t-test p-value**			**Effect Size (Cohen's d)**			**Wilcoxon rank-sum p-value**			**Lilliefors Test p-value**		
	**CL-VNS vs. Rehab**	**CL-VNS vs. Delayed VNS**	**Rehab vs. Delayed VNS**	**CL-VNS vs. Rehab**	**CL-VNS vs. Delayed VNS**	**Rehab vs. Delayed VNS**	**CL-VNS vs. Rehab**	**CL-VNS vs. Delayed VNS**	**Rehab vs. Delayed VNS**	**CL-VNS**	**Delayed VNS**	**Rehab**
**PRV GFP Total**	0.0169	N/A	N/A	2.067	N/A	N/A	0.0043	N/A	N/A	0.0522	N/A	0.35
**PRV RFP Total**	0.817	N/A	N/A	0.158	N/A	N/A	0.9	N/A	N/A	0.24	N/A	0.5
**PRV Double Labeled (%)**	0.0013	N/A	N/A	3.443	N/A	N/A	0.0158	N/A	N/A	0.394	N/A	0.5
**Myelin Area - Proximal**	0.774	0.619	0.3	0.304	0.59	1.11	0.7	0.7	0.4	N/A	N/A	N/A
**Myelin Area - Distal**	0.675	0.258	0.495	0.368	1.081	0.618	0.7	0.4	0.7	N/A	N/A	N/A
**Muscle Fiber Area**	0.689	0.601	0.474	0.22	0.31	0.47	0.832	0.694	0.876	0.5	0.5	0.045

	**Student's t-test p-value**			**Effect Size (Cohen's d)**			**Wilcoxon rank-sum p-value**			**Lilliefors Test p-value**		
	**CL-VNS vs. Rehab**	**CL-VNS vs. ACh:-/CL-**	**Rehab vs. ACh:-/CL-VNS**	**CL-VNS vs. Rehab**	**CL-VNS vs. ACh:-/CL-**	**Rehab vs. ACh:-/CL-VNS**	**CL-VNS vs. Rehab**	**CL-VNS vs. ACh:-/CL-**	**Rehab vs. ACh:-/CL-VNS**	**CL-VNS**	**ACh:-/CL-VNS**	**Rehab**

		VNS			VNS			VNS				
**ICMS Digit Flexion**	0.092	0.0015	0.0041	1.441	5.459	4.479	0.2	0.028	0.028	0.5	0.001	0.5
**ICMS Extension**	0.0321	0.00054	0.0015	2.771	6.689	4.368	0.142	0.028	0.028	0.001	0.5	0.5

	**Student's t-test p-value**			**Effect Size (Cohen's d)**			**Wilcoxon rank-sum p-value**			**Lilliefors Test p-value**		
**<u>Success Rate</u>**	**CL-VNS vs. Rehab**	**CL-VNS vs. ACh:-/CL-VNS**	**Rehab vs. ACh:-/CL-VNS**	**CL-VNS vs. Rehab**	**CL-VNS vs. ACh:-/CL-VNS**	**Rehab vs. ACh:-/CL-VNS**	**CL-VNS vs. Rehab**	**CL-VNS vs. ACh:-/CL-VNS**	**Rehab vs. ACh:-/CL-VNS**	**CL-VNS**	**ACh:-/CL-VNS**	**Rehab**
**Pre**	0.9199	0.7087	0.6777	0.0482	0.2014	0.224	0.8633	0.1898	0.1898	0.4553	0.0492	0.5
**Week 6**	0.2628	0.3532	0.3753	0.7239	0.8797	0.8387	0.3267	0.7912	0.2517	0.001	0.001	0.001
**Week 7**	0.956	0.3314	0.2985	0.0264	0.9097	0.9765	0.9585	0.5315	0.5874	0.001	0.0784	0.001
**Week 8**	0.0543	0.074	0.3138	1.093	1.7647	0.93	0.0547	0.0979	0.8881	0.0985	0.3742	0.001
**Week 9**	0.011	0.0174	0.4206	1.4308	2.4013	0.6964	0.014	0.027	0.9001	0.4625	0.4685	0.0027
**Week 10**	0.0056	0.0021	0.6064	1.5092	2.4951	0.3473	0.0077	0.004	0.8252	0.2786	0.0958	0.0022
**Week 11**	0.008	0.0004	0.7828	1.5676	2.5539	0.1758	0.0503	0.004	0.6993	0.5	0.3803	0.003
**Week 12**	0.0007	0.0001	0.2528	2.2018	6.1082	0.848	0.004	0.001	0.5185	0.5	0.4884	0.038

	**Student's t-test p-value**			**Effect Size (Cohen's d)**			**Wilcoxon rank-sum p-value**			**Lilliefors Test p-value**		
**<u>Pull Force</u>**	**CL-VNS vs. Rehab**	**CL-VNS vs. ACh:-/CL-VNS**	**Rehab vs. ACh:-/CL-VNS**	**CL-VNS vs. Rehab**	**CL-VNS vs. ACh:-/CL-VNS**	**Rehab vs. ACh:-/CL-VNS**	**CL-VNS vs. Rehab**	**CL-VNS vs. ACh:-/CL-VNS**	**Rehab vs. ACh:-/CL-VNS**	**CL-VNS**	**ACh:-/CL-VNS**	**Rehab**
**Pre**	0.897	0.1308	0.1172	0.062	0.8594	0.8965	0.7962	0.1469	0.2977	0.5	0.1033	0.0641
**Week 6**	0.6193	0.2735	0.4993	0.239	0.8125	0.4867	0.2973	0.2398	0.7223	0.1671	0.1005	0.0396
**Week 7**	0.1208	0.0511	0.5977	0.7725	1.5616	0.3887	0.0503	0.0599	0.6993	0.5	0.5	0.001
**Week 8**	0.0083	0.0177	0.775	1.4498	1.8871	0.1832	0.0142	0.019	1	0.5	0.3911	0.2122
**Week 9**	0.0064	0.0036	0.549	1.4773	2.5747	0.4329	0.0078	0.004	0.8981	0.5	0.5	0.5

**Week 10**	0.0014	0.0005	0.9353	1.865	2.6971	0.0526	0.004	0.002	0.8981	0.3983	0.5	0.337
**Week 11**	0.0174	0.0011	0.9838	1.4575	2.252	0.013	0.0625	0.004	0.7972	0.5	0.5	0.25
**Week 12**	0.0036	0.0001	0.237	1.8362	4.3322	0.7884	0.0078	0.001	0.4376	0.5	0.5	0.1283

	**Student's t-test p-value**			**Effect Size (Cohen's d)**			**Wilcoxon rank-sum p-value**			**Lilliefors Test p-value**		
	**CL-VNS vs. Rehab**	**CL-VNS vs. ACh:-/CL-VNS**	**Rehab vs. ACh:-/CL-VNS**	**CL-VNS vs. Rehab**	**CL-VNS vs. ACh:-/CL-VNS**	**Rehab vs. ACh:-/CL-VNS**	**CL-VNS vs. Rehab**	**CL-VNS vs. ACh:-/CL-VNS**	**Rehab vs. ACh:-/CL-VNS**	**CL-VNS**	**ACh:-/CL-VNS**	**Rehab**
**Von Frey Right**	0.0197	0.00126	0.808	1.938	3.073	0.165	0.0317	0.0079	1	0.5	0.5	0.5

Supplementary Table 20. Statistical tests for all figures.

ID	Group	Cuff Impedance (KOhms)
1	CL-VNS	7.6
2	CL-VNS	4.3
3	CL-VNS	4.8
4	CL-VNS	8
5	CL-VNS	4.1
6	CL-VNS	5.5
7	CL-VNS	6.1
8	CL-VNS	8.9
9	CL-VNS	8.4
10	CL-VNS	4.5
24	Delayed VNS	3.3
25	Delayed VNS	8.1
26	Delayed VNS	3.8
27	Delayed VNS	6.4
28	Delayed VNS	7.2
29	Delayed VNS	7.7
30	Delayed VNS	3.6
31	Delayed VNS	4.3
32	Delayed VNS	4.9
33	Delayed VNS	4
34	Delayed VNS	6.9
35.00	CL-VNS	6.1
36.00	CL-VNS	4.2
37.00	CL-VNS	8.1
38.00	CL-VNS	3.1
39.00	CL-VNS	3.3
40.00	CL-VNS	7.4
41.00	CL-VNS	5.7
42.00	CL-VNS	5.9
43.00	CL-VNS	3.9
53.00	ACh-:CL-VNS	3.1
54.00	ACh-:CL-VNS	5.9
55.00	ACh-:CL-VNS	5.1

56.00	ACh-:CL-VNS	8.1
57.00	ACh-:CL-VNS	3.5

Supplementary Table 21. Cuff impedance for subjects at Week 12.
