# Supplementary material for: Enhancing plasticity in central networks improves motor and sensory recovery after nerve damage
Source: Nat Commun. 2019 Dec 19;10:5782. doi: 10.1038/s41467-019-13695-0 (PMC6923364; doi:10.1038/s41467-019-13695-0)
Supplement: Supplementary file 3 — Description of Additional Supplementary Files [file 41467_2019_13695_MOESM3_ESM.pdf]

Description of Additional Supplementary Files

File Name: Supplementary Movie 1
Description: *Pull task performance prior to nerve damage*
Representative example of a rat performing the isometric pull task prior to median and ulnar nerve damage. Note that the rat is highly proficient at the task and displays excellent forelimb motor control.

The plot on the bottom left displays the pull force of the manipulandum plotted in real time for an individual trial. The horizontal green line indicates the force threshold to trigger delivery of a reward pellet.

File Name: Supplementary Movie 2
Description: *Pull task performance six weeks after median and ulnar nerve damage*
Representative example of a rat performing the isometric pull task six weeks after damage to the median and ulnar nerves. The rat displays a notable reduction in peak pull forces, consistent with a reduction in forelimb strength, and clear sensorimotor deficits.

The plot on the bottom left displays the pull force of the manipulandum plotted in real time for an individual trial. The horizontal green line indicates the force threshold to trigger delivery of a reward pellet.

File Name: Supplementary Movie 3
Description: *Pull task performance after seven weeks of rehabilitation*
Representative example of a rat performing the isometric pull task following seven weeks of rehabilitative training and thirteen weeks after damage to the median and ulnar nerves. A notable reduction in peak pull forces are still apparent despite seven weeks of daily rehabilitative training.

The plot on the bottom left displays the pull force of the manipulandum plotted in real time for an individual trial. The horizontal green line indicates the force threshold to trigger delivery of a reward pellet.

File Name: Supplementary Movie 4
Description: *Pull task performance after seven weeks of rehabilitation with VNS*
Representative example of a rat performing the isometric pull task following seven weeks of rehabilitative training with VNS. Note the improvements in motor control and increases in force production compared to rehabilitative training alone (Supplementary Video 3).

The plot on the bottom left displays the pull force of the manipulandum plotted in real time for an individual trial. The horizontal green line indicates the force threshold to trigger delivery of a reward pellet and VNS. Vertical red lines above the force traces indicate VNS pulse train delivery immediately when the pull force exceeds the stimulation threshold.
