# Supplementary material for: Enhancing plasticity in central networks improves motor and sensory recovery after nerve damage
Source: Nat Commun. 2019 Dec 19;10:5782. doi: 10.1038/s41467-019-13695-0 (PMC6923364; doi:10.1038/s41467-019-13695-0)
Supplement: Supplementary file 8 — Reporting Summary [file 41467_2019_13695_MOESM8_ESM.pdf]

nature research

Corresponding author(s): Eric Meyers

Last updated by author(s): Oct 30, 2019

Reporting Summary

Nature Research wishes to improve the reproducibility of the work that we publish. This form provides structure for consistency and transparency in reporting. For further information on Nature Research policies, see Authors & Referees and the Editorial Policy Checklist.

Statistics

For all statistical analyses, confirm that the following items are present in the figure legend, table legend, main text, or Methods section.

n/a	Confirmed	
☐	☒	The exact sample size (*n*) for each experimental group/condition, given as a discrete number and unit of measurement
☐	☒	A statement on whether measurements were taken from distinct samples or whether the same sample was measured repeatedly
☐	☒	The statistical test(s) used AND whether they are one- or two-sided<br>*Only common tests should be described solely by name; describe more complex techniques in the Methods section.*
☐	☒	A description of all covariates tested
☐	☒	A description of any assumptions or corrections, such as tests of normality and adjustment for multiple comparisons
☐	☒	A full description of the statistical parameters including central tendency (e.g. means) or other basic estimates (e.g. regression coefficient) AND variation (e.g. standard deviation) or associated estimates of uncertainty (e.g. confidence intervals)
☐	☒	For null hypothesis testing, the test statistic (e.g. *F*, *t*, *r*) with confidence intervals, effect sizes, degrees of freedom and *P* value noted<br>*Give P values as exact values whenever suitable.*
☒	☐	For Bayesian analysis, information on the choice of priors and Markov chain Monte Carlo settings
☒	☐	For hierarchical and complex designs, identification of the appropriate level for tests and full reporting of outcomes
☐	☒	Estimates of effect sizes (e.g. Cohen's *d*, Pearson's *r*), indicating how they were calculated

Our web collection on statistics for biologists contains articles on many of the points above.

Software and code

Policy information about availability of computer code

Data collection	Behavioral data was collected using the MotoTrak Pull system (http://www.vulintus.com/mototrak)
Data analysis	All data analysis was performed in MATLAB 2016b

For manuscripts utilizing custom algorithms or software that are central to the research but not yet described in published literature, software must be made available to editors/reviewers. We strongly encourage code deposition in a community repository (e.g. GitHub). See the Nature Research guidelines for submitting code & software for further information.

Data

Policy information about availability of data

All manuscripts must include a data availability statement. This statement should provide the following information, where applicable:

- Accession codes, unique identifiers, or web links for publicly available datasets
- A list of figures that have associated raw data
- A description of any restrictions on data availability

The datasets generated during and/or analyzed during the current study are available from the corresponding author on reasonable request. Electron microscopy images are available at the following links: 10.6084/m9.figshare.8239751, 10.6084/m9.figshare.8239694, 10.6084/m9.figshare.8239754.

Field-specific reporting

Please select the one below that is the best fit for your research. If you are not sure, read the appropriate sections before making your selection.

☒ Life sciences ☐ Behavioural & social sciences ☐ Ecological, evolutionary & environmental sciences

Life sciences study design

All studies must disclose on these points even when the disclosure is negative.

Sample size	Sample sizes were chosen based on previous studies in stroke models using VNS to enhance recovery of motor function (described in Methods).
Data exclusions	49 rats were excluded from this study based upon the following exclusion criteria: (1) Did not survive the peripheral nerve injury surgical procedure or VNS cuff implant (N=15); (2) Did not display at least a 50% reduction in maximal pull force (N=6); (3) Too impaired to freely perform the task following peripheral nerve injury (N=9); (4) Headcap or stimulating cuff failure (N=19). 30 of the 49 exclusions (1-3) were done prior to group assignment and thus could not impact interpretation of results.
Replication	An internal replication of the main behavioral effect of CL-VNS to improve motor function was performed in and described in detail in Experiment 2.
Randomization	Rats were dynamically allocated to balanced groups based on Week 6 maximal pull forces. All allocations were performed prior to treatment.
Blinding	Experimenters were blind to the treatment group during testing and analysis.

Reporting for specific materials, systems and methods

We require information from authors about some types of materials, experimental systems and methods used in many studies. Here, indicate whether each material, system or method listed is relevant to your study. If you are not sure if a list item applies to your research, read the appropriate section before selecting a response.

Materials & experimental systems

n/a	Involved in the study
☒	☐ Antibodies
☒	☐ Eukaryotic cell lines
☒	☐ Palaeontology
☐	☒ Animals and other organisms
☒	☐ Human research participants
☒	☐ Clinical data

Methods

n/a	Involved in the study
☒	☐ ChIP-seq
☒	☐ Flow cytometry
☒	☐ MRI-based neuroimaging

Animals and other organisms

Policy information about studies involving animals; ARRIVE guidelines recommended for reporting animal research

Laboratory animals	106 adult (~3 months) female Sprague-Dawley rats were studied, each weighing approximately 250g at the start of the study.
Wild animals	The study did not involve wild animals.
Field-collected samples	The study did not involve samples collected from the field.
Ethics oversight	All handling, housing, surgical procedures, and behavioral training were approved by The University of Texas at Dallas Institutional Animal Care and Use Committee.

Note that full information on the approval of the study protocol must also be provided in the manuscript.
